# MRI‐based radiomic signatures for pretreatment prognostication in cervical cancer

**DOI:** 10.1002/cam4.6526

**Published:** 2023-10-16

**Authors:** Kari S. Wagner‐Larsen, Erlend Hodneland, Kristine E. Fasmer, Njål Lura, Kathrine Woie, Bjørn I. Bertelsen, Øyvind Salvesen, Mari K. Halle, Noeska Smit, Camilla Krakstad, Ingfrid S. Haldorsen

**Affiliations:** ^1^ Mohn Medical Imaging and Visualization Centre (MMIV), Department of Radiology Haukeland University Hospital Bergen Norway; ^2^ Section for Radiology, Department of Clinical Medicine University of Bergen Bergen Norway; ^3^ Department of Mathematics University of Bergen Bergen Norway; ^4^ Department of Obstetrics and Gynecology Haukeland University Hospital Bergen Norway; ^5^ Department of Pathology Haukeland University Hospital Bergen Norway; ^6^ Clinical Research Unit, Department of Clinical and Molecular Medicine Norwegian University of Science and Technology Trondheim Norway; ^7^ Centre for Cancer Biomarkers (CCBIO), Department of Clinical Science University of Bergen Bergen Norway; ^8^ Department of Informatics University of Bergen Bergen Norway

**Keywords:** biomarkers, magnetic resonance imaging, prognostication, radiomics, uterine cervical neoplasms

## Abstract

**Background:**

Accurate pretherapeutic prognostication is important for tailoring treatment in cervical cancer (CC).

**Purpose:**

To investigate whether pretreatment MRI‐based radiomic signatures predict disease‐specific survival (DSS) in CC.

**Study Type:**

Retrospective.

**Population:**

CC patients (*n* = 133) allocated into training_(T)_ (*n*_T_ = 89)/validation_(V)_ (*n*_V_ = 44) cohorts.

**Field Strength/Sequence:**

T2‐weighted imaging (T2WI) and diffusion‐weighted imaging (DWI) at 1.5T or 3.0T.

**Assessment:**

Radiomic features from segmented tumors were extracted from T2WI and DWI (high *b*‐value DWI and apparent diffusion coefficient (ADC) maps).

**Statistical Tests:**

Radiomic signatures for prediction of DSS from T2WI (T2_rad_) and T2WI with DWI (T2 + DWI_rad_) were constructed by least absolute shrinkage and selection operator (LASSO) Cox regression. Area under time‐dependent receiver operating characteristics curves (AUC) were used to evaluate and compare the prognostic performance of the radiomic signatures, MRI‐derived maximum tumor size ≤/> 4 cm (MAX_size_), and 2018 International Federation of Gynecology and Obstetrics (FIGO) stage (I–II/III–IV). Survival was analyzed using Cox model estimating hazard ratios (HR) and Kaplan–Meier method with log‐rank tests.

**Results:**

The radiomic signatures T2_rad_ and T2 + DWI_rad_ yielded AUC_T_/AUC_V_ of 0.80/0.62 and 0.81/0.75, respectively, for predicting 5‐year DSS. Both signatures yielded better or equal prognostic performance to that of MAX_size_ (AUC_T_/AUC_V_: 0.69/0.65) and FIGO (AUC_T_/AUC_V_: 0.77/0.64) and were significant predictors of DSS after adjusting for FIGO (HR_T_/HR_V_ for T2_rad_: 4.0/2.5 and T2 + DWI_rad_: 4.8/2.1). Adding T2_rad_ and T2 + DWI_rad_ to FIGO significantly improved DSS prediction compared to FIGO alone in cohort_(T)_ (AUC_T_ 0.86 and 0.88 vs. 0.77), and FIGO with T2 + DWI_rad_ tended to the same in cohort_(V)_ (AUC_V_ 0.75 vs. 0.64, *p* = 0.07). High radiomic score for T2 + DWI_rad_ was significantly associated with reduced DSS in both cohorts.

**Data Conclusion:**

Radiomic signatures from T2WI and T2WI with DWI may provide added value for pretreatment risk assessment and for guiding tailored treatment strategies in CC.

## Graphical abstract

Whole‐volume radiomic tumor profiling from T2WI and DWI may contribute to pretherapeutic non‐invasive prognostication in cervical cancer. The radiomic signatures derived from T2WI alone and both T2WI and DWI yielded moderate‐to‐high diagnostic performance for predicting disease‐specific survival (DSS), and demonstrated equal or better prognostic performance to that of MRI‐derived maximum tumor size ≤/> 4 cm and 2018 FIGO stage (I–II/III–IV). Adding the radiomic signatures to 2018 FIGO stage (I–II/III–IV) improved DSS prediction compared to FIGO alone in the training cohort, and the combination of FIGO with T2 + DWI_rad_ tended to the same in the validation cohort.

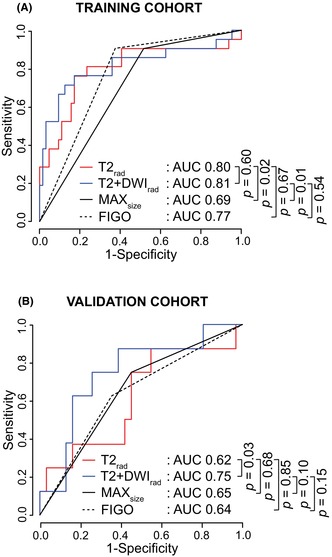

## 1 INTRODUCTION

Cervical cancer (CC) represents a major global health challenge. It is the most common gynecologic malignancy and the fourth leading cause of cancer‐related death in women worldwide.^1^ CCs are staged according to the 2018 International Federation of Gynecology and Obstetrics (FIGO) system, which guides the stratification of patients to different treatment regimens. Surgery is the gold standard for the management of early‐stage disease (FIGO IA–IB2), while for patients with large (>4 cm) tumors or locally advanced disease (FIGO IB3–IVA), concurrent chemoradiation is the recommended treatment.^2^ Five‐year survival is highly affected by stage, ranging from 92% in early‐stage disease to 58% in locally advanced disease, and 18% in metastatic disease.^3^

Major prognostic factors in CC include FIGO stage, tumor size, and lymph node involvement.^4^, ^5^ Other well‐known factors affecting survival are histological subtype and grade, deep stromal invasion, lymphovascular space invasion (LVSI), and patient age.^6^, ^7^, ^8^ Importantly, extensive histopathologic assessments of primary tumor characteristics and spread, such as deep stromal invasion, LVSI, and lymph node metastases, can only be obtained through examination of surgical specimens, which is only available in patients with presumed early‐stage disease who receive primary surgery. Clinical non‐invasive tools that provide refined tumor characterization and prognostication before start of therapy may guide more targeted therapeutic strategies and thereby improve patient outcome. Thus, there is an urgent need to develop non‐invasive pretherapeutic biomarkers to identify high‐risk patients and guide tailored and targeted treatments in CC.

Given its excellent soft tissue resolution, magnetic resonance imaging (MRI) has long been considered the imaging method of choice for assessing local tumor extent in CC.^9^ T2‐weighted imaging (T2WI) visualizes detailed anatomical structures, whereas diffusion‐weighted imaging (DWI) depicts functional tissue properties that may be highly different in benign and malignant tissue.^10^ The apparent diffusion coefficients (ADC) derived from DWI yield quantitative assessments of water diffusion properties of the tumor, indirectly reflecting the tumor microenvironment. Low tumor ADC values have been linked to reduced survival in CC.^11^ However, quantitative metrics obtained from MRI are usually presented as mean tumor values in selected regions of interest (ROIs), which inherently are incapable of capturing whole‐volume features reflecting, for example, tumor heterogeneity.

Radiomic tumor profiling involves the extraction of large‐scale quantitative imaging features based on radiological images.^12^ The radiomic features are typically invisible to the naked eye but may reveal tumor characteristics linked to clinical phenotype and prognosis.^13^ As such, radiomics allows non‐invasive tumor profiling that may capture intratumoral complexity and heterogeneity.^14^ Studies on CC have linked MRI tumor radiomics to FIGO stage and histopathological markers.^15^, ^16^, ^17^, ^18^, ^19^, ^20^, ^21^ Furthermore, MRI‐based radiomic signatures have recently been recognized as valuable biomarkers for predicting recurrence and survival in CC patients.^22^, ^23^, ^24^, ^25^, ^26^

The purpose of this study was to investigate whether pretreatment MRI radiomic whole‐volume tumor profiling based on T2WI and DWI may aid in prognostication in CC. Furthermore, we aimed to compare the prognostic performance of radiomic signatures with conventional clinical markers and explore the potential added value of radiomic signatures for guiding therapeutic strategy in CC.

## 2 MATERIALS AND METHODS

### 2.1 Patients and study setting

**FIGURE 1:**
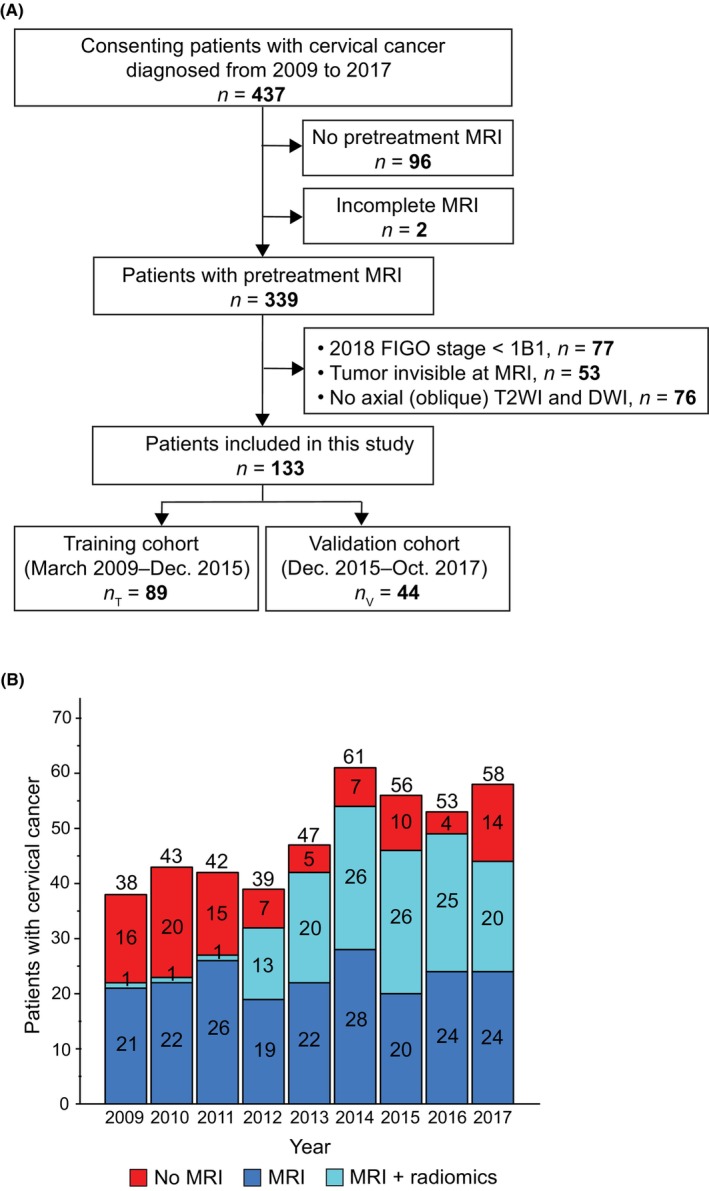
(A) Study flowchart. The study cohort is based on a cohort of consenting patients (participation rate in the order of >95%) with histologically verified cervical cancer diagnosed from 2009 to 2017 at our hospital who were subjected to pretreatment pelvic MRI. The included patients (*n* = 133) in this study were diagnosed with 2018 FIGO stage ≥IB1, had visible tumor on MRI, and an imaging protocol that included axial (oblique) T2‐weighted imaging (T2WI) and diffusion‐weighted imaging (DWI). (B) Bar graph depicting the number of cervical cancer patients diagnosed each year during the same period (2009–2017; *n* = 437), showing the subgroups of patients without pretreatment MRI (*n* = 98; red); and of patients with pretreatment MRI (*n* = 339; blue and light blue), among whom 133 patients (light blue) were eligible for MRI radiomic tumor profiling.

This retrospective study on prospectively collected data was approved by the Regional Committee for Medical Research Ethics (2015/2333/REK vest) with written informed consent at primary diagnosis from all patients. Patients admitted to the hospital with histologically verified CC and a complete, pretreatment MRI from May 2009 to December 2017 (*n* = 339) were enrolled (Figure 1). Inclusion criteria were: (I) 2018 FIGO stage ≥IB1, (II) visible tumor on T2WI confirmed by two radiologists, and (III) the MRI protocol included axial/axial oblique (perpendicular to the long axis of the cervix) T2WI and axial/axial oblique DWI. A total of 133 CC patients met the inclusion criteria and were allocated in a 2:1 ratio to a training cohort_(T)_ (*n*_T_ = 89, diagnosed from March 2009 to December 2015) and a validation cohort_(V)_ (*n*_V_ = 44, diagnosed from December 2015 to October 2017) according to the time the MRI was acquired (Figure 1).

Clinical‐ and histopathological patient data (e.g., age, menopausal status, histologic tumor type and grade, and primary treatment) was retrieved from medical records. All patients were retrospectively restaged according to the 2018 FIGO criteria.^27^ Conventional radiological staging parameters, including maximum tumor size measured irrespective of plane on T2WI, were recorded as previously described.^28^

Compared with the entire MRI CC cohort (*n* = 339), patients in the radiomics cohort (*n* = 133) were older, had larger MRI‐derived maximum tumor size, higher 2018 FIGO stage, and more frequently received radiotherapy ± chemotherapy (*p* < 0.05 for all) (Table S1).

Date of last follow‐up was August 2022. Disease‐specific survival (DSS) was defined as time from primary diagnosis until death caused by CC. By last follow‐up, 26% (23/89) of the patients in cohort_(T)_  and 23% (10/44) in cohort_(V)_ had died from CC (Table 1). Median (mean) [interquartile range, IQR] follow‐up for survivors in cohort_(T)_/cohort_(V)_ was 102 (103) [91–112]/70 (69) [64–73] months.

**TABLE 1:** Clinical‐ and pathological patient characteristics for the 133 cervical cancer patients included in the study; and separate figures for the training cohort (*n*_T_ = 89; diagnosed March 2009–December 2015), and the validation cohort (*n*_V_ = 44; diagnosed December 2015–October 2017).

	Radiomics cohort (*n* = 133)	Training cohort (*n*_T_ = 89)	Validation cohort (*n*_V_ = 44)	*p*
Age, median (IQR) (*n* = 133)	48 (37–60)	46 (36–60)	49 (45–59)	0.15
BMI, kg/m^2^, median (IQR) (*n* = 133)	26 (22–28)	26 (22–29)	24 (22–27)	0.38

	*n* (%)	*n*_T_ (%)	*n*_V_ (%)	
Menopausal status (*n* = 130)				0.34
Pre‐/perimenopausal	74 (57)	53 (60)	21 (50)	
Postmenopausal	56 (43)	35 (40)	21 (50)	
MRI‐derived maximum tumor size (*n* = 133)				0.23
≤2 cm	19 (14)	10 (11)	9 (20)	
>2 and ≤4 cm	38 (29)	24 (27)	14 (32)	
>4 cm	76 (57)	55 (62)	21 (48)	
2018 FIGO stage (*n* = 133)				0.55
I	41 (31)	25 (28)	16 (36)	
II	30 (23)	19 (21)	11 (25)	
III	47 (35)	35 (39)	12 (27)	
IV	15 (11)	10 (11)	5 (11)	
Histologic type (*n* = 133)				0.89
Squamous cell carcinoma	104 (78)	69 (78)	35 (80)	
Adenocarcinoma	21 (16)	15 (17)	6 (14)	
Other^a^	8 (6)	5 (6)	3 (7)	
Histologic grade (*n* = 124)				0.62
1&2	102 (82)	66 (81)	36 (86)	
3	22 (18)	16 (20)	6 (14)	
Primary treatment (*n* = 133)				0.39
Surgery only^b^	34 (26)	19 (21)	15 (34)	
Surgery^c^ and adjuvant treatment^d^	12 (9)	8 (9)	4 (9)	
Radiotherapy ± chemotherapy	79 (59)	57 (64)	22 (50)	
Other^e^	8 (6)	5 (6)	3 (7)	
Dead from cervical cancer (*n* = 133)				0.83
Yes	33 (25)	23 (26)	10 (23)	
No	100 (75)	66 (74)	34 (77)	

*Note*: *p* values refer to test of differences between the training‐ and validation cohort (Wilcoxon rank‐sum test for continuous variables and Fisher's exact test for categorical variables). Significant *p* values are given in bold.

Abbreviations: BMI, body mass index; FIGO, International Federation of Gynecology and Obstetrics; IQR, interquartile range.

^a^ Adenosquamous (*n* = 2), neuroendocrine (*n* = 4), or undifferentiated carcinomas (*n* = 2).

^b^ Hysterectomy ± bilateral salpingectomy/salpingo‐oophorectomy.

^c^ Conization (*n* = 1), trachelectomy (*n* = 1), or hysterectomy ± bilateral salpingectomy/salpingo‐oophorectomy (*n* = 10).

^d^ Chemoradiation combined (*n* = 10) or chemotherapy only (*n* = 2).

^e^ Neoadjuvant chemotherapy followed by surgery (*n* = 1) or palliative treatment (*n* = 7).

### 2.2 MRI scanning

Pretreatment pelvic MRI, performed as part of routine clinical workup, was acquired on scanners from different vendors (GE Healthcare, USA; Siemens Healthineers, Germany; Philips Healthcare, Netherlands), comprising 1.5T (96/133 patients) or 3.0T (37/133 patients) systems at three different hospitals in Western Norway. The imaging protocols and scanning parameters varied across scanners and institutions. However, all examinations were dedicated pelvic protocols, mainly in accordance with the European Society of Urogenital Radiology (ESUR) guidelines for staging of CC.^9^ Pelvic axial and/or axial oblique, sagittal, and coronal and/or coronal oblique (parallel to the long axis of the cervix) T2WI, axial T1‐weighted imaging (T1WI), and axial and/or axial oblique DWI (*b*‐values 0/50–800/1000 s/mm^2^) were performed for all patients. A detailed overview of MRI acquisition parameters is listed in Table S2. The MRI examinations in cohort_(T)_ and cohort_(V)_ had similar distributions in terms of institutions and vendors used. Cohort_(T)_ comprised mainly 1.5T examinations (1.5T 92%; 82/89 vs. 3.0T 8%; 7/89), whereas cohort_(V)_ were mostly 3T examinations (1.5T 32%; 14/44 vs. 3.0T 68%; 30/44).

### 2.3 Tumor segmentation

**FIGURE 2:**
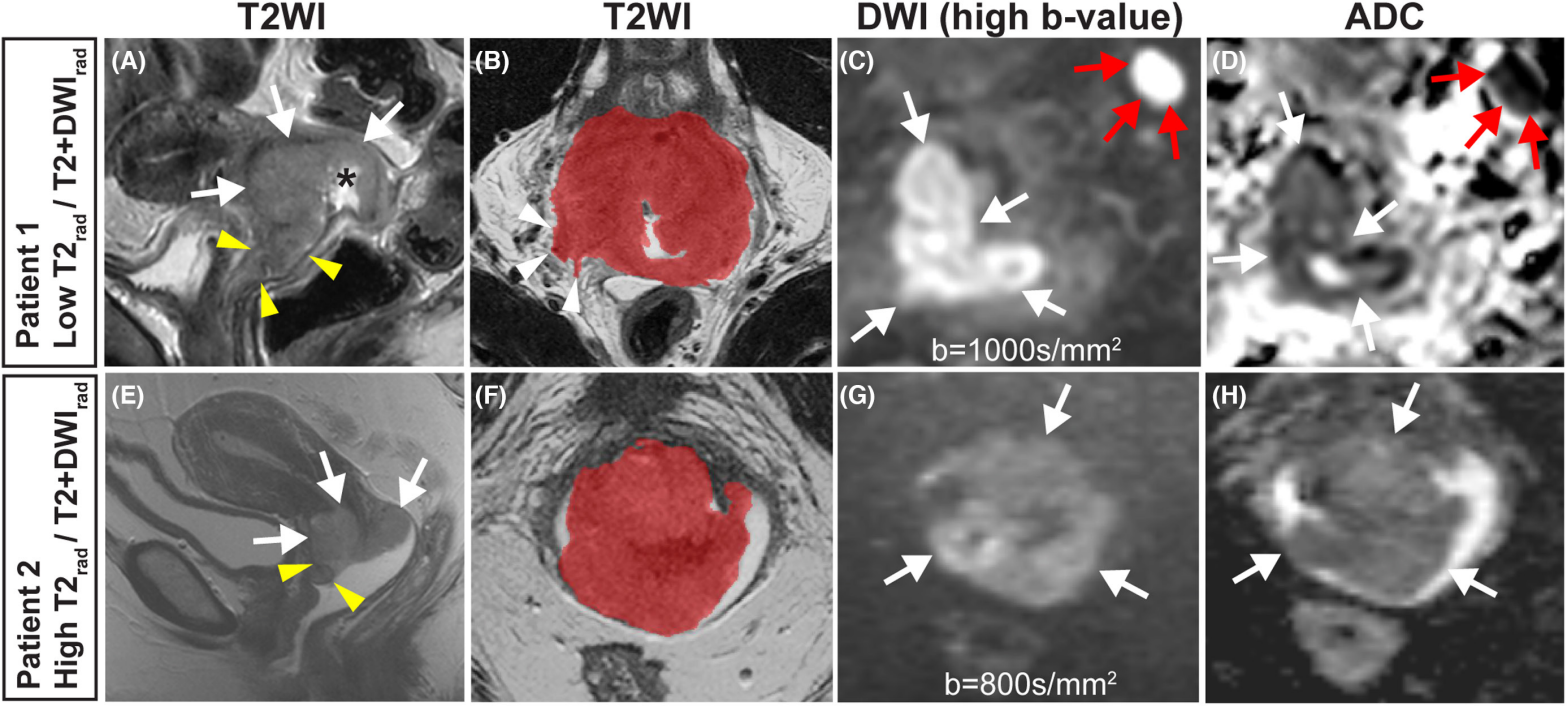
Cervical cancer depicted by sagittal T2‐weighted imaging (T2WI) (A, E), axial oblique T2WI (with manually segmented tumor mask) (B, F), axial/axial oblique diffusion‐weighted imaging (DWI) (*b* = 1000 s/mm^2^ and *b* = 800 s/mm^2^) (C, G) with corresponding apparent diffusion coefficient (ADC) maps (D, H) in two different patients diagnosed with 2018 FIGO stage IIIC1. (A–D) Patient 1: A 48‐year‐old woman having low radiomic score for the signatures T2_rad_ and T2 + DWI_rad_ and a large cervical tumor (white arrows; squamous cell carcinoma) with central ulceration (A, black asterisk). MRI‐measured maximum tumor size is 5.9 cm. The tumor extends to the upper 1/3 of the vagina (A, yellow arrowheads) and invades the parametrium (B, white arrowheads). An enlarged pelvic lymph node (short axis diameter 1.2 cm) is depicted with restricted diffusion (C, D red arrows). The primary tumor also exhibits restricted diffusion (white arrows) with hyperintensity on high *b*‐value (C), and hypointensity on the corresponding ADC map (D). The patient was subjected to primary chemoradiotherapy with no signs of recurrence 5.5 years after treatment. (E–H) Patient 2: A 47‐year‐old woman having high radiomic score for both T2_rad_ and T2 + DWI_rad_ and a large cervical tumor (white arrows; adenocarcinoma). MRI‐measured maximum tumor size is 4.8 cm, and the tumor extends to the upper part of the vagina (E, yellow arrowheads). The primary tumor exhibits restricted diffusion (white arrows) with hyperintensity on high *b*‐value (G), and hypointensity on the corresponding ADC map (H). The patient also had an enlarged pelvic lymph node (short axis diameter 1.2 cm; not shown) with restricted diffusion close to the left external iliac vessels. The patient received primary chemoradiotherapy, but experienced lymph node recurrence and died from cervical cancer 3.5 years after treatment.

CC primary lesions were segmented manually on axial oblique (when available) or axial T2WI on all slices depicting tumor (Figure 2B,F). The open‐source software ITK‐SNAP (version 3.6.0, www.itksnap.org ^29^) was used for the tumor segmentation. DWI images and contrast‐enhanced T1WI (CE T1WI) (performed in 14/133 patients) were available for visual inspection to verify tumor borders. The tumor segmentations were conducted by one radiologist in 106 cases (Reader 1 [K.W.L]: *n* = 59; Reader 2 [N.L]: *n* = 47), and by both radiologists in 27 randomly chosen cases. The two radiologists had 12 and 7 years of experience reading pelvic MRIs and segmented the tumors independently and blinded for clinicopathological patient information. The two readers demonstrated good agreement on the manual tumor segmentations with a median [IQR] Dice score of 0.81 [0.73–0.86]. The extracted tumor masks were exported in the Neuroimaging Informatics Technology Initiative (NIfTI) file format.^30^

### 2.4 Image resampling

For each patient, the DWI images (high *b*‐value DWI and ADC maps) were resampled using trilinear interpolation to the same slice thickness and voxel size as the corresponding axial (oblique) T2WI used for tumor segmentation. After transformation, image voxel data for each patient were specified on the same spatial grid. Out‐of‐grid extrapolation values were set to zero.

### 2.5 Feature extraction

Image data and tumor masks were loaded using the Imagedata library.^31^ Before feature extraction, the T2WI and DWI series were normalized to obtain a standardized distribution of image voxel intensities. For this normalization, each data *f* set was divided by its own average, computed for voxels greater than zero, and then multiplied by a factor of 100;

$$f\leftarrow f∙\frac{100}{{\bar{f}}_{f>0}}$$

Radiomic feature extraction was conducted using the Image Biomarkers Standardization Initiative (IBSI)^32^ compliant PyRadiomics package for Python.^33^ Radiomic features were extracted from the T2WI axial (oblique) images and from the DWI images (high *b*‐value DWI and ADC maps) that were resampled on the T2WI axial (oblique) grid. The position of the tumor masks on each slice of the resampled DWI series was validated by a radiologist (Reader 1).

A total of 292 radiomic features of primary tumor were extracted for each patient; 106 from T2WI (including 13 from the tumor mask), 93 from high *b*‐value DWI, and 93 and from ADC maps. The radiomic features included shape‐based features (*n* = 13), first‐order statistical features (*n* = 54), and textural features (*n* = 225). Textural features comprised five classes: 72 Gray Level Co‐occurrence Matrix (GLCM), 42 Gray Level Dependence Matrix (GLDM), 48 Gray Level Run Length Matrix (GLRLM), 48 Gray Level Size Zone Matrix (GLSZM), and 15 Neighboring Gray Tone Difference Matrix (NGTDM). The extracted features were z‐normalized according to each vendor and field strength, except for shape‐based features that were z‐normalized across the entire dataset. The shape‐based features were calculated from the tumor mask, thus largely independent of vendor and field strength.

### 2.6 Feature selection and radiomic signature construction

A two‐step procedure was applied for radiomic feature selection. First, the robustness of the radiomic features extracted from the segmentations performed by both radiologists was assessed by intraclass correlation coefficients (ICCs). Radiomic features with ICC >0.75 were considered reliable and retained for further analysis. This resulted in a radiomic dataset consisting of 206 features: 60 features from T2WI, 77 from high *b*‐value DWI, and 69 from ADC (Table S3). Second, least absolute shrinkage and selection operator (LASSO) Cox regression^34^ and Elastic net Cox regression^35^ for prediction of DSS were applied for radiomic feature selection and radiomic signature development in cohort_(T)_. Both methods were utilized to assess the optimal approach for feature selection in this dataset. The regularization parameter λ (LASSO Cox and Elastic net Cox) was optimized by leave‐one‐out cross‐validation, and the regularization parameter α (Elastic net Cox) was set to 0.5.

Radiomic signatures were constructed with the selected features from T2WI only (T2_rad_) and from both T2WI and the DWI series (high *b*‐value DWI and ADC map) (T2 + DWI_rad_), respectively. Radiomic scores were then calculated by a linear combination of final selected significant features multiplied by their respective coefficients.

Elastic net Cox selected a larger number of radiomic features than LASSO Cox; however, this did not improve the prognostic performance of the radiomic signatures (Table S4). Thus, LASSO Cox, representing a simpler model with fewer radiomic features, was used for further analyses.

### 2.7 Model performance assessment

The radiomic signatures were derived from cohort_(T)_ and tested in cohort_(V)_. All subsequent analyses were done separately for the two respective cohorts. The performance of the radiomic signatures for predicting 5‐year DSS was assessed and compared with MRI‐measured maximum tumor diameter ≤/> 4 cm (MAX_size_) and 2018 FIGO stage (I–II/III–IV) using area under time‐dependent receiver operating characteristic (tdROC) analyses (AUC). Harrell's concordance index (C‐index) for overall DSS was calculated for further discrimination of prognostic performance of the two radiomic signatures. The value of the radiomic signatures for predicting DSS was compared with that of standard clinicopathological variables (FIGO stage, MRI‐derived tumor size, age, and histologic grade) using the Cox proportional hazards model. All variables satisfied the assumption of proportional hazard (the Schoenfeld test of residuals and graphical diagnostics), except histologic grade (1 & 2 vs. 3) in the cohort_(V)_ (*p <* 0.05). To determine whether radiomic signatures combined with FIGO stage and MAX_size_ could improve DSS prediction, the significant predictors of DSS in the univariable Cox analysis in cohort_(T)_ were used to build models combining radiomic signatures and FIGO stage (I–II/III–IV)/MAX_size_: FIGO with T2_rad_, FIGO with T2 + DWI_rad_, MAX_size_ with T2_rad,_ and MAX_size_ with T2 + DWI_rad_. The models were evaluated by AUC from tdROC analysis, nested likelihood ratio test, and Akaike information criterion (AIC).

Optimal cutoff values for the radiomic signatures T2_rad_ and T2 + DWI_rad_ derived in cohort_(T)_ were identified from tdROC curves using Youden Index, and the patients were subsequently divided into high (high‐risk)‐ and low (low‐risk) radiomic score groups. Differences in DSS between the groups were explored using the Kaplan–Meier method with the log‐rank tests.

### 2.8 Statistical analyses

Statistical analyses were conducted using R 4.2.1 (R Core Team, 2022^36^) and STATA 17.0 (StataCorp. 2021^37^). Differences in clinicopathological characteristics between cohort_(T)_ versus cohort_(V)_ and the radiomics cohort versus the entire MRI CC cohort were assessed using the Wilcoxon rank sum test for continuous variables and the Fisher's exact test for categorical variables, respectively. LASSO Cox and Elastic net Cox regression were implemented using the “glmnet” R‐package. The “timeROC” R‐package was used for calculating AUC for DSS at 5 years. The “survivalROC” R‐package was adopted for determining the optimal cutoff values for the tdROC curves in the training cohort using Youden Index. Spearman's rank correlation coefficient measured the correlation between the selected radiomic features in the signatures T2_rad_ and T2 + DWI_rad_. All reported *p* values were generated by two‐sided tests and considered significant when <0.05.

## 3 RESULTS

### 3.1 Clinical characteristics

The median [IQR] age at primary diagnosis for the patient cohort (*n* = 133) was 48 [37–60] years. Altogether, 53% (71/133) were diagnosed with 2018 FIGO stage I–II and 47% (62/133) with stage III–IV. The clinical‐ and pathological characteristics of the patients are shown in Table 1. There was no significant difference in clinicopathological parameters or DSS between cohort_(T)_ and cohort_(V)_ (*p* = 0.15–0.89) (Table 1).

### 3.2 Radiomic signature construction

The constructed radiomic signature from the Lasso Cox regression model for T2WI only (T2_rad_) included one GLSZM‐ and three shape‐based features. The radiomic signature from both T2WI and DWI (T2 + DWI_rad_) included three GLSZM‐, one shape‐based‐, and one GLCM feature (Table 2). Details of the radiomic signature formulas and radiomic score calculation are described in Appendix S1.

**TABLE 2:** Radiomic feature coefficients selected by LASSO Cox regression from T2WI alone and T2WI in combination with DWI for prediction of disease‐specific survival (DSS) in cervical cancer. The regularization parameter (λ) was optimized by leave‐one‐out cross‐validation. The radiomic signatures were derived from the training cohort (*n*_T_ = 89) and tested in the validation cohort (*n*_V_ = 44). The performance metrics of the radiomic signatures for predicting DSS are given as AUC (5‐year DSS) and C‐index (overall DSS).

Radiomic features	Radiomic signature T2_rad_ ^a^	Radiomic signature T2 + DWI_rad_ ^b^
GLSZM large area low gray level emphasis T2WI	0.188	0.276
Shape major axis length T2WI	0.333	
Shape maximum 2D diameter slice T2WI	0.003	
Shape surface area T2WI	0.093	0.291
GLCM cluster shade DWI (high *b*‐value)		0.001
GLSZM size zone non‐uniformity DWI (high *b*‐value)		0.173
GLSZM gray level non‐uniformity ADC		0.276
Regularization parameter λ	0.112	0.097

Metrics for prognostic performance	Radiomic signature T2_rad_ ^a^	Radiomic signature T2 + DWI_rad_ ^b^
(*n*_T_ = 89/*n*_V_ = 44)	LASSO Cox	LASSO Cox
AUC_T_	0.80	0.81
AUC_V_	0.62	0.75
C‐index_T_	0.73	0.76
C‐index_V_	0.63	0.72

Abbreviations: ADC, apparent diffusion coefficient; AUC, area under the time‐dependent receiver operating characteristic (tdROC) curves; C‐Index, concordance index; DWI, diffusion‐weighted imaging; GLCM, gray level co‐occurrence matrix; GLSZM, gray level size zone matrix; LASSO, least absolute shrinkage and selection operator; T2WI, T2‐weighted imaging.

^a^ Radiomic features derived only from T2WI.

^b^ Radiomic features derived from T2WI and DWI (high *b*‐value and ADC).

There were moderate‐to‐strong positive correlations between the radiomic features in T2_rad_ (*r*_S_ = 0.51–0.94, *p* < 0.05 for all) (Table S5). In T2 + DWI_rad_, the radiomic features were only weak to moderately correlated (*r*_S_ = −0.07–0.10 for the non‐significant correlations, *p* ≥ 0.23 for all; *r*_S_ = 0.38–0.58 for the significant correlations, *p* < 0.05 for all), except for Shape Surface Area T2WI and GLSZM Gray Level Non‐Uniformity ADC, which were strongly correlated (*r*_S_ = 0.78, *p* < 0.05) (Table S6).

### 3.3 Prognostic performance of the radiomic signatures

**FIGURE 3:**
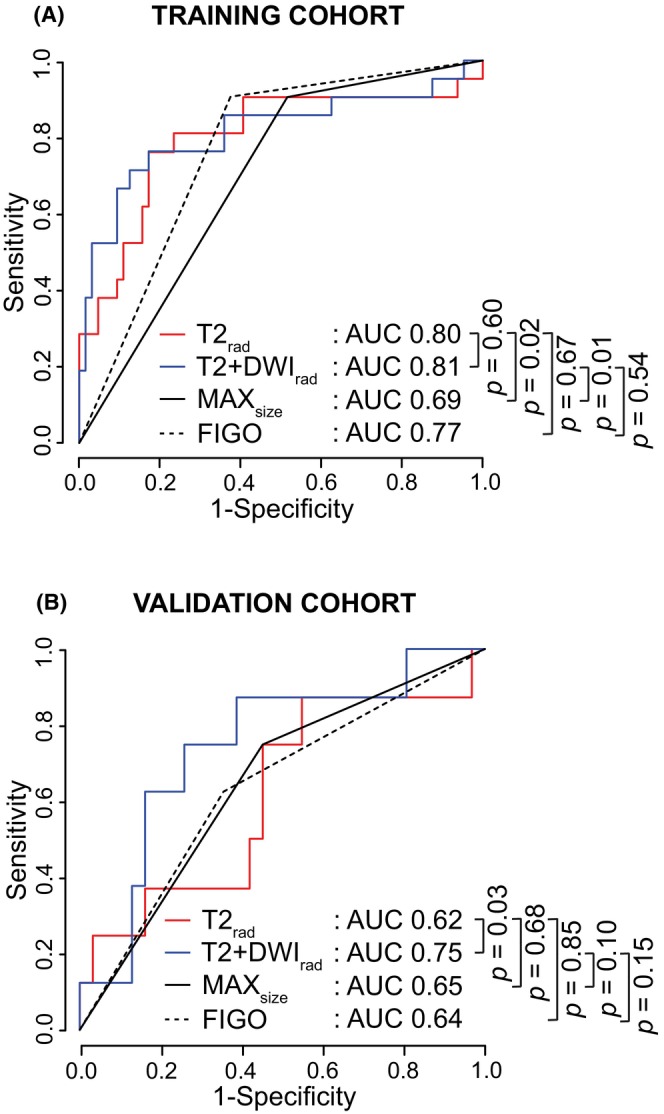
Time‐dependent receiver operating characteristic (tdROC) curves for prediction of 5‐year disease‐specific survival (DSS) based on the radiomic signature from T2WI only (T2_rad_), from both T2WI and DWI (T2 + DWI_rad_), MRI‐measured maximum tumor size ≤/> 4 cm (MAX_size_), and 2018 FIGO stage (I–II/III–IV) in the training (*n*_T_ = 89) (A) and validation (*n*_V_ = 44) (B) cohorts. *p* values refer to the test of equal area under the tdROC curves (AUC). FIGO, International Federation of Gynecology and Obstetrics.

The tdROC curves for predicting 5‐year DSS yielded AUC_T_/AUC_V_ of 0.80/0.62 for T2_rad_, 0.81/0.75 for T2 + DWI_rad_, 0.69/0.65 for MAX_size_, and 0.77/0.64 for FIGO (Figure 3 and Table 2). In cohort_(T)_, T2_rad_ and T2 + DWI_rad_ yielded significantly higher AUC_T_ than MAX_size_ (*p* < 0.05 for both) but similar AUC_T_ to FIGO (*p* = 0.67 and *p* = 0.54) (Figure 3A). In cohort_(V)_, T2 + DWI_rad_ tended to yield higher AUC than MAX_size_ and FIGO (*p* = 0.10 and *p* = 0.15, respectively) (Figure 3B). While T2_rad_ and T2 + DWI_rad_ yielded similar performance metrics in cohort_(T)_ (*p* = 0.60), T2 + DWI_rad_ outperformed T2_rad_ in the cohort_(V)_ (*p* < 0.05) (Figure 3). T2 + DWI_rad_ also yielded higher C‐indexes than T2_rad_ in both cohorts (C‐index_T_/C‐index_V_ for T2 + DWI_rad_ and T2_rad_ of 0.76/0.72 and 0.73/0.63, respectively) (Table 2).

T2_rad_ and T2 + DWI_rad_ significantly predicted DSS (HR_T_/HR_V_ of 5.3/2.8 and 5.8/2.2, respectively; *p* < 0.05 for all) (Table 3). Furthermore, when adjusting for FIGO stage (I–II/III–IV), the radiomic signatures remained independent predictors of DSS in both cohorts (HR_T_/HR_V_ of 4.0/2.5 and 4.8/2.1; *p* < 0.05 for all). Also when adjusting for MAX_size_, both signatures remained significantly associated with reduced survival in cohort_(T)_ (HR_T_: 4.6 and 5.7; *p* < 0.05 for both) and tended to the same in the cohort_(V)_ (HR_V_: 2.1 and 1.7; *p* = 0.09 and *p* = 0.14, respectively) (Table 3).

**TABLE 3:** Radiomic signatures (T2_rad_ and T2 + DWI_rad_), 2018 FIGO stage, MRI‐derived maximum tumor size ≤/>4 cm (MAX_size_), patient age, and histologic grade for predicting disease‐specific survival (DSS) in the training (*n*_T_ = 89) and the validation (*n*_V_ = 44) cohorts.

	Univariable HR (95% CI)	*p* ^a^	Multivariable: T2_rad_ and FIGO HR (95% CI)	*p* ^a^	Multivariable: T2 + DWI_rad_ and FIGO HR (95% CI)	*p* ^a^	Multivariable: T2_rad_ and MAX_size_ HR (95% CI)	*p* ^a^	Multivariable: T2 + DWI_rad_ and MAX_size_ HR (95% CI)	*p* ^a^
Training cohort										
T2_rad_ (*n* = 89)	5.3 (2.8–10.1)	**<0.001**	4.0 (2.1–7.9)	**<0.001**			4.6 (2.3–9.2)	**<0.001**		
T2 + DWI_rad_ (*n* = 89)	5.8 (3.2–10.6)	**<0.001**			4.8 (2.5–9.1)	**<0.001**			5.7 (2.9–11.1)	**<0.001**
FIGO stage (I–II/III–IV) (*n* = 89)	5.9 (2.0–17.5)	**0.001**	4.1 (1.3–12.4)	**0.01**	3.2 (1.0–9.8)	**0.04**				
MAX_size_ (*n* = 89)	3.7 (1.3–10.8)	**0.02**					1.8 (0.6–5.9)	0.30	1.1 (0.3–3.8)	0.89
Age at prim. diag., per decade (*n* = 89)	1.2 (1.0–1.6)	0.10								
Histologic grade (1 & 2 vs.3) (*n* = 82)	1.9 (0.8–5.0)	0.17								
Validation cohort										
T2_rad_ (*n* = 44)	2.8 (1.3–5.8)	**0.007**	2.5 (1.1–5.6)	**0.03**			2.1 (0.9–5.1)	0.09		
T2 + DWI_rad_ (*n* = 44)	2.2 (1.2–3.9)	**0.006**			2.1 (1.1–4.0)	**0.04**			1.7 (0.8–3.6)	0.14
FIGO stage (I–II/III–IV) (*n* = 44)	2.5 (0.7–8.9)	0.16	1.6 (0.4–6.4)	0.52	1.3 (0.3–5.9)	0.73				
MAX_size_ (*n* = 44)	4.9 (1.0–23.3)	**0.04**					3.2 (0.6–16.8)	0.18	2.8 (0.5–16.6)	0.26
Age at prim. diag., per decade (*n* = 44)	1.4 (1.0–2.1)	0.08								
Histologic grade (1&2 vs. 3) (*n* = 42)	3.2 (0.8–12.6)	0.10								

Abbreviations: CI, confidence interval; FIGO, International Federation of Gynecology and Obstetrics; HR, hazard ratio.

^a^ Cox regression analysis; significant *p* values are given in bold.

### 3.4 Prognostic performance of the combined models

**FIGURE 4:**
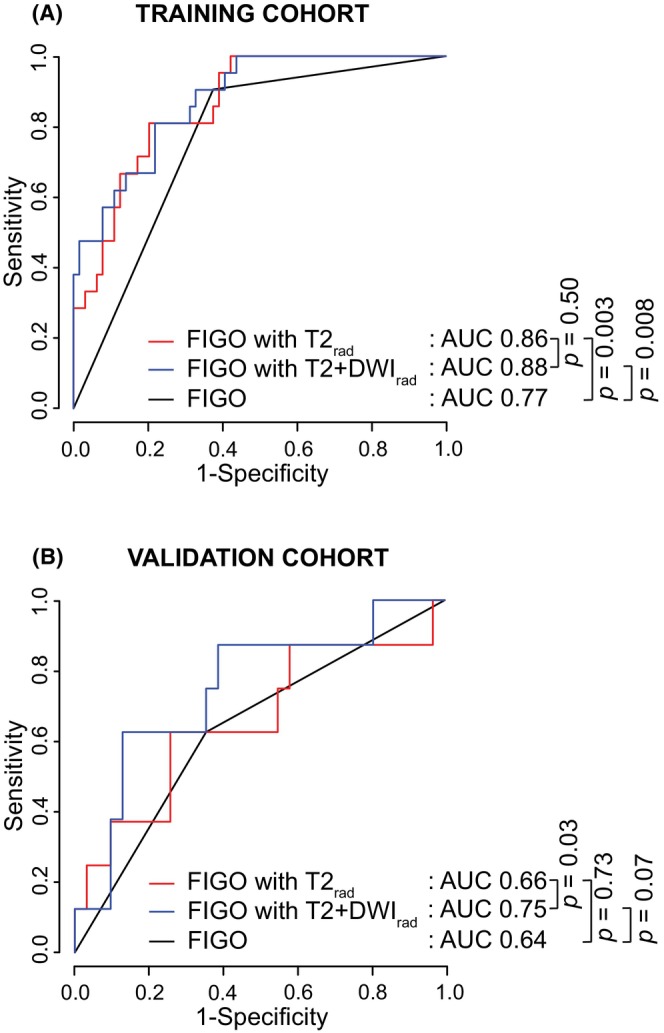
Time‐dependent receiver operating characteristic (tdROC) curves for prediction of 5‐year disease‐specific survival (DSS) based on 2018 FIGO stage (I–II/III–IV), a combined model of FIGO with T2_rad_, and a combined model of FIGO with T2 + DWI_rad_ in the training (*n*_T_ = 89) (A) and validation (*n*_V_ = 44) (B) cohorts. *p* values refer to the test of equal area under the tdROC curves (AUC). FIGO, International Federation of Gynecology and Obstetrics.

For the combined radiomic‐ and FIGO model the tdROC analysis for prediction of 5‐year DSS yielded AUC_T_/AUC_V_ of 0.86/0.66 for FIGO with T2_rad_, 0.88/0.75 for FIGO with T2 + DWI_rad_, and 0.77/0.64 for FIGO alone (Figure 4 and Table 4). Both FIGO‐radiomic models performed significantly better than FIGO alone (*p* < 0.05 for both) in cohort_(T)_, while FIGO with T2 + DWI_rad_ showed a strong tendency to the same in cohort_(V)_ (*p* = 0.07) (Figure 4). Whereas FIGO with T2 + DWI_rad_ and FIGO with T2_rad_ yielded similar prognostic performance in cohort_(T)_ (*p* = 0.50), FIGO with T2 + DWI_rad_ outperformed FIGO with T2_rad_ in cohort_(V)_ (*p* < 0.05) (Figure 4).

**TABLE 4:** The performance of combined 2018 FIGO‐radiomic models and 2018 FIGO stage alone for predicting 5‐year disease‐specific survival (DSS) in cervical cancer.

	Training cohort (*n*_T_ = 89)		Validation cohort (*n*_V_ = 44)	
	AUC_T_	AIC_T_	AUC_V_	AIC_V_
FIGO with T2_rad_	0.86	175.3	0.66	68.0
FIGO with T2 + DWI_rad_	0.88	167.3	0.75	67.9
FIGO	0.77	186.3	0.64	69.6

Abbreviations: AUC, area under the time‐dependent receiver operating characteristic (tdROC) curves; AIC, akaike information criterion; FIGO, International Federation of Gynecology and Obstetrics.

The FIGO‐radiomic models had a better model fit than FIGO alone in cohort_(T)_ as demonstrated by nested likelihood ratio test (*p* < 0.05 for both models) and lower AIC_T_ (175.3 and 167.3 for FIGO with T2_rad_, FIGO with T2 + DWI_rad_ vs. 186.3 for FIGO), with a similar tendency in cohort_(V)_ (*p* = 0.06 for both models and corresponding AIC_V_ of 68.0 and 67.9 vs. 69.6) (Table 4).

Similarly, the combined MAX_size_‐radiomic models mostly yielded higher AUCs than MAX_size_ alone; however, without a significant improvement in model fit in cohort_(V)_ (Figure S1 and Table S7).

### 3.5 Prediction of survival in low‐ and high‐radiomic score groups

**FIGURE 5:**
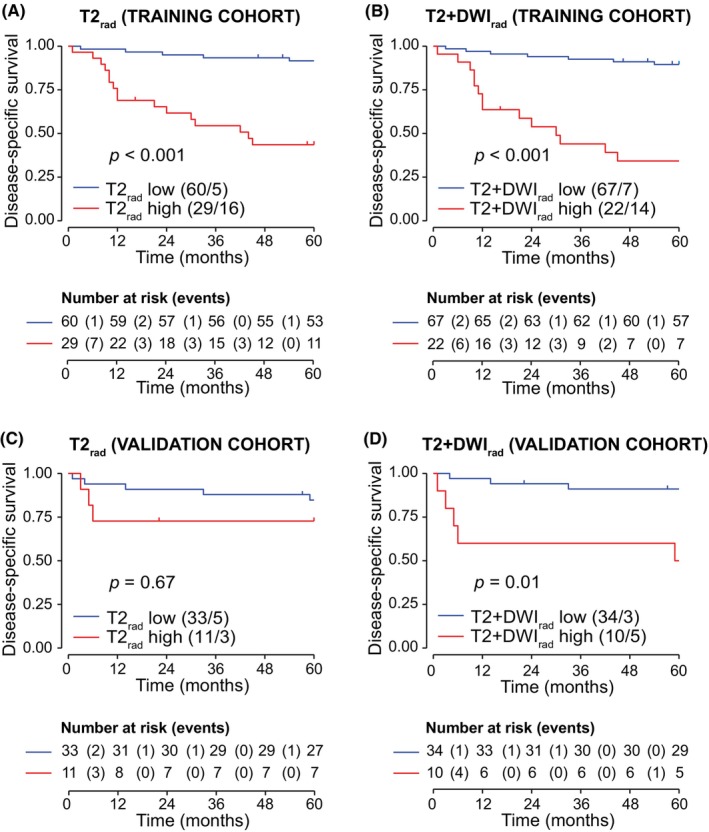
Kaplan–Meier curves depicting disease‐specific survival (DSS) in cervical cancer patients with low‐ and high‐radiomic score for the signatures T2_rad_ and T2 + DWI_rad_ in the training (*n*_T_ = 89) (A and B) and validation (*n*_V_ = 44) (C and D) cohorts. The optimal cutoff values for the radiomic signatures were identified from time‐dependent receiver operating characteristics (tdROC) curve analysis in the training cohort using Youden Index.

Higher radiomic score for T2_rad_ and T2 + DWI_rad_ was associated with reduced DSS in cohort_(T)_ (*p* < 0.05 for both). In cohort_(V)_, patients with high T2 + DWI_rad_ score demonstrated worse DSS (*p* < 0.05), whereas no significant difference in survival was observed between low‐ and high‐score groups for T2_rad_ (*p* = 0.67) (Figure 5). Moreover, patients with high T2_rad_ and T2 + DWI_rad_ radiomic scores were more often diagnosed with local or distant tumor progression than patients with low radiomic scores (Table S8). Imaging findings in two patients having similar 2018 FIGO stage (IIIC1) but displaying different low‐risk/high‐risk radiomic scores are presented in Figure 2.

## 4 DISCUSSION

This study demonstrates that whole‐volume radiomic tumor profiling from T2WI and DWI may contribute to pretherapeutic non‐invasive prognostication in CC. The developed radiomic signatures based on T2WI (T2_rad_) alone and both T2WI and DWI (T2 + DWI_rad_) yielded moderate‐to‐high diagnostic performance for predicting DSS. Importantly, both radiomic signatures demonstrated better or equal prognostic performance to that of MRI‐derived maximum tumor size ≤/> 4 cm (MAX_size_) and 2018 FIGO stage (I–II/III–IV). Combining radiomic signatures with FIGO stage yielded higher DSS prediction accuracy than FIGO stage alone. MRI radiomic tumor profiling may represent a promising supplement to conventional MRI staging information allowing refined pretreatment prognostication and treatment tailoring in CC.

In CC, MRI radiomic signatures have previously been shown to predict high‐risk clinicopathological features, that is, parametrial‐ or deep stromal tumor invasion,^15^, ^16^ LVSI,^17^ high histologic grade,^18^ and lymph node metastases^19^, ^20^, ^21^ in subgroups of patients receiving primary surgical treatment. Furthermore, MRI‐based CC radiomic tumor profiles have been linked to increased risk of recurrence or death in a few recent studies.^22^, ^23^, ^24^, ^25^, ^26^ The latter studies (comprising 183–248 patients) report moderate‐to‐high performance metrics for predicting disease‐free/progression‐free survival based on radiomic signatures from various combinations of MRI‐sequences (T2WI, DWI, and CE T1WI). Their reported AUCs (training/validation cohort) are in the range of 0.73–0.86/0.66–0.81^22^, ^23^, ^24^ and C‐indexes (training/validation cohort) in the range of 0.74–0.79/0.67–0.81.^23^, ^24^, ^25^, ^26^ In the present study, the achieved prognostic performance metrics of the radiomic signatures (AUCs for 5‐year DSS for T2_rad_: 0.80/0.62 and T2 + DWI_rad_: 0.81/0.75; C‐indexes for T2_rad_: 0.73/0.63 and T2 + DWI_rad_: 0.76/0.72) are broadly in line with that previously reported. However, large methodological variations exist among MRI studies, that is, differences in MRI sequences, field strength, patient demographics, approaches for radiomic feature extraction, and statistical methods. This makes direct comparison of reported performance metrics from radiomic models difficult as the impact of methodological variation is not fully known. Importantly, the potential clinical utility, robustness, and reproducibility of radiomic tumor profiling for prognostication in CC need to be tested and validated in independent CC cohorts at different centers prior to implementation in the clinic.

Currently, MRI‐derived tumor size and 2018 FIGO stage are routinely used in the clinic to guide choice of therapy in CC.^2^ To the best of our knowledge, this is the first study to comprehensively evaluate the predictive ability of MRI radiomic signatures with rigorous separation of the training‐ and validation cohorts in all analyses, in comparison and combination with these conventional markers. Interestingly, the radiomic signatures in the present study yielded better or similar prognostic performance to that of MRI‐derived maximum tumor size ≤/> 4 cm (MAX_size_) and 2018 FIGO stage (I–II/III–IV). Furthermore, the radiomic signatures remained independent predictors of DSS even after adjusting for 2018 FIGO stage (both in cohort_(T)_ and cohort_(V)_) and after adjusting for MAX_size_ (in cohort_(T)_). This emphasizes the possible benefit of combining radiomic signatures and conventional staging markers to enhance the prediction of pretreatment prognosis in CC.

We found that T2 + DWI_rad_ outperformed T2_rad_ for predicting 5‐year DSS in cohort_(V)_, suggesting that DWI radiomic features may capture biologically relevant features not fully captured by T2WI, which are important for prognosis. In line with this, two previous studies on early‐stage CC report that combined T2WI‐ and DWI radiomic signatures outperform T2WI signatures for predicting parametrial invasion^15^ and lymph node metastases^19^; both being surrogate markers of poor prognosis. Similarly, a recent study of CC patients by Zheng et al., reports that a combined T2WI‐ and DWI radiomic signature yields better prediction of 3‐year disease‐free survival than the T2WI signature (AUCs of 0.83/0.77 vs. 0.73/0.66 in the training/validation cohorts) (*n* = 207).^22^ However, Zheng et al. included only patients diagnosed with early‐stage disease at a single center using 3.0T MRI,^22^ whereas our study included patients with all FIGO stages ≥IB1, diagnosed at multiple centers with 1.5T and 3.0T MRI systems. Despite these differences, the overall similar findings support a likely benefit of a combined T2WI and DWI radiomic tumor profile for prognostication in CC.

The 2018 revision of the FIGO classification system for CC allows including clinical‐, radiological‐, or pathological findings in stage assignment.^2^ The 2018 FIGO stage is strongly linked to prognosis as it incorporates key factors such as tumor size, parametrial invasion, lymph node metastases, and distant spread, all known to impact survival. Importantly, the final 2018 FIGO stage is based on a combination of many examinations, all of which are inherently susceptible to subjectivity, and variability. An objective and accurate supplement to FIGO for prognostication of CC would therefore be of clinical value. In the present study, we found that the prognostic models combining radiomic profiles (T2_rad_ and T2 + DWI_rad_) and 2018 FIGO stage (I–II/III–IV) yielded better prediction of survival and better model fit than that of FIGO stage alone. Interestingly, a previous study reports that pretherapeutic computed tomography (CT) radiomic tumor signatures in combination with 2009 FIGO stage (IB1, II, III, IVA) yields better prediction of survival and better model fit than FIGO stage alone in CC (*n* = 106).^38^ The added value of radiomic tumor profiling as a supplement to FIGO stage for better prognostication in CC may hence be shared by both CT‐ and MRI radiomic profiling. However, this needs to be validated in larger and independent patient cohorts.

Of note, the primary tumor features captured by MRI radiomic prognostic profiling do not have a unique histopathologic correlate or an obvious pathogenic interpretation. However, increased intratumoral heterogeneity, known to be associated with aggressive tumor biology and resistance to therapy in CC,^39^ may putatively be reflected in radiomic features. Thus, whole‐volume radiomic tumor profiling has the potential, by providing non‐invasive markers of tissue heterogeneity, to yield whole‐volume tumor heterogeneity markers beyond what is accessible from that of tumor biopsies.^14^ Furthermore, with increasing knowledge about likely links between radiomic tumor profiles and potential molecular targets for treatment, radiomic tumor profiling may enable more tailored, and targeted treatment strategies in future CC patient care.

### 4.1 Limitations

First, the MRI examinations were conducted in a multicenter setting with scanners from different vendors comprising both 1.5T and 3.0T systems using a variety of image protocols, which might have influenced the image quality, tumor segmentation accuracy, and reproducibility of the radiomic feature extraction. However, one could argue that this approach to a certain extent reduces the risk of selection bias and demonstrates the robustness of predictive models. As such, our results may be generalizable and reflect the real‐world use of MRI‐based radiomics in clinical practice. Based on recommendations in the literature, radiomic features with an ICC ≤0.75 from segmentations conducted by both radiologists were excluded.^14^ This criteria for radiomic feature selection may reportedly be suboptimal.^40^ Further investigation into the repeatability and stability of MRI radiomic features for prognostic modeling is clearly warranted. Second, the tumor masks were delineated manually on axial (oblique) T2WI and placed on resampled DWI. While a radiologist validated the accuracy of the tumor mask placement on each slice of the DWI series, it would have been ideal that the primary tumor segmentations were drawn specifically on these series. However, manual tumor segmentation on high *b*‐value DWI and ADC maps in addition to T2WI would be extremely time‐consuming and certainly not feasible in a clinical radiology workflow. Thus, the need to develop new software applications that allow automated and accurate tumor segmentations on multiparametric MRI is crucial if future advancements in radiomic profiling are to be introduced in the clinic. Third, to avoid overfitting, we used the recommended temporal split method when allocating patients into cohorts.^41^ This splitting resulted in the training cohort comprising mainly 1.5T examinations and the validation cohort primarily 3.0T examinations. Thus, since the models were trained using older MRI systems and validated in newer MRI systems having better imaging technologies, this may have partly influenced the diagnostic performance of the radiomic models. Fourth, our study encompassed patients who underwent varied treatments based on their disease stage, potentially influencing patient outcomes. Unfortunately, our relatively small study cohort precluded an assessment of how the radiomic signatures differed for the various treatment groups. However, future larger radiomic studies should ideally include analyses for the different treatment groups separately. Lastly, the robustness of our results remains to be externally validated in large and independent CC cohorts prior to potential implementation in the clinic.

## 5 CONCLUSION

The radiomic signatures derived from T2WI only (T2_rad_) and both T2WI and DWI (T2 + DWI_rad_) based on pretreatment MRI demonstrated moderate‐to‐high diagnostic performance for predicting DSS in CC. Both radiomic signatures performed better or similar to that based on MRI‐derived maximum tumor size (≤/>4 cm) and 2018 FIGO stage (I–II/III–IV). Adding the radiomic signatures to 2018 FIGO stage (I–II/III–IV) improved DSS prediction compared to FIGO alone in the training cohort, and the combination of FIGO with T2 + DWI_rad_ tended to the same in the validation cohort. Furthermore, our findings suggest a likely advantage of using combined T2WI‐ and DWI radiomic tumor signatures over T2WI signatures for pretreatment risk assessment in CC. This study supports the promising role of radiomic tumor profiling for refined pretreatment prognostication and for guiding tailored treatment strategies in CC.

## AUTHOR CONTRIBUTIONS

**Kari S. Wagner‐Larsen:** Conceptualization (equal); data curation (lead); formal analysis (lead); funding acquisition (equal); investigation (lead); methodology (equal); project administration (equal); resources (supporting); software (supporting); validation (equal); visualization (equal); writing – original draft (lead); writing – review and editing (lead). **Erlend Hodneland:** Data curation (equal); formal analysis (equal); investigation (supporting); methodology (supporting); software (equal); validation (supporting); writing – original draft (supporting); writing – review and editing (supporting). **Kristine E. Fasmer:** Conceptualization (supporting); data curation (supporting); formal analysis (supporting); investigation (supporting); methodology (supporting); writing – original draft (supporting). **Njål Lura:** Data curation (supporting); formal analysis (supporting); methodology (supporting); writing – original draft (supporting). **Kathrine Woie:** Data curation (supporting); writing – original draft (supporting). **Bjørn Inge Bertelsen:** Data curation (supporting); writing – original draft (supporting). **Oyvind Salvesen:** Conceptualization (supporting); formal analysis (supporting); methodology (supporting); validation (supporting); writing – original draft (supporting); writing – review and editing (supporting). **Mari Kylleso Halle:** Data curation (supporting); writing – original draft (supporting). **Noeska Smit:** Visualization (supporting); writing – original draft (supporting). **Camilla Krakstad:** Conceptualization (supporting); data curation (supporting); formal analysis (supporting); funding acquisition (supporting); methodology (supporting); supervision (supporting); writing – original draft (supporting). **Ingfrid S Haldorsen:** Conceptualization (equal); data curation (supporting); formal analysis (supporting); funding acquisition (equal); investigation (supporting); methodology (equal); project administration (equal); resources (lead); supervision (lead); validation (equal); visualization (equal); writing – original draft (supporting); writing – review and editing (supporting).

## FUNDING INFORMATION

This project was funded by the Trond Mohn Foundation (BFS2018TMT06), the Western Norway Regional Health Authority (grant number F‐12168), and the Norwegian Research Council (grant number 311350).

## CONFLICT OF INTEREST STATEMENT

The authors of this manuscript declare no relationships with any companies, whose products or services may be related to the subject matter of the article.

All authors had access to all the data in the study and accept responsibility to submit for publication.

## ETHICS STATEMENT

Institutional Review Board approval was obtained: IRB approval#: 2015/2333/REK vest.

## PATIENS CONSENT STATEMENT

Written informed consent was obtained at primary diagnosis from all subjects in this retrospective study on prospectively collected data.

## Supporting information


Data S1.

## Data Availability

The data that support the findings of this study are available on request from the corresponding author. The data are not publicly available due to privacy or ethical restrictions.

## References

[1] Sung H , Ferlay J , Siegel RL , et al. Global cancer statistics 2020: GLOBOCAN estimates of incidence and mortality worldwide for 36 cancers in 185 countries. CA Cancer J Clin. 2021;71:209‐249. 33538338 10.3322/caac.21660

[2] Bhatla N , Aoki D , Sharma DN , Sankaranarayanan R . Cancer of the cervix uteri: 2021 update. Int J Gynaecol Obstet. 2021;155 Suppl 1(Suppl 1):28‐44. 34669203 10.1002/ijgo.13865 PMC9298213

[3] Siegel RL , Miller KD , Fuchs HE , Jemal A . Cancer statistics, 2022. CA Cancer J Clin. 2022;72(1):7‐33. 35020204 10.3322/caac.21708

[4] Matsuo K , Machida H , Mandelbaum RS , Konishi I , Mikami M . Validation of the 2018 FIGO cervical cancer staging system. Gynecol Oncol. 2019;152(1):87‐93. 30389105 10.1016/j.ygyno.2018.10.026 PMC7528458

[5] Wright JD , Matsuo K , Huang Y , et al. Prognostic performance of the 2018 International Federation of Gynecology and Obstetrics cervical cancer staging guidelines. Obstet Gynecol. 2019;134(1):49‐57. 31188324 10.1097/AOG.0000000000003311 PMC7641496

[6] Cohen PA , Jhingran A , Oaknin A , Denny L . Cervical cancer. Lancet. 2019;393(10167):169‐182. 30638582 10.1016/S0140-6736(18)32470-X

[7] Waggoner SE . Cervical cancer. Lancet. 2003;361(9376):2217‐2225. 12842378 10.1016/S0140-6736(03)13778-6

[8] Halle MK , Ojesina AI , Engerud H , et al. Clinicopathologic and molecular markers in cervical carcinoma: a prospective cohort study. Am J Obstet Gynecol. 2017;217(4):432.e431‐432.e417. 10.1016/j.ajog.2017.05.068 28599900

[9] Manganaro L , Lakhman Y , Bharwani N , et al. Staging, recurrence and follow‐up of uterine cervical cancer using MRI: Updated Guidelines of the European Society of Urogenital Radiology after revised FIGO staging 2018. Eur Radiol. 2021;31:7802‐7816. 33852049 10.1007/s00330-020-07632-9

[10] Bollineni VR , Kramer G , Liu Y , Melidis C , deSouza NM . A literature review of the association between diffusion‐weighted MRI derived apparent diffusion coefficient and tumour aggressiveness in pelvic cancer. Cancer Treat Rev. 2015;41(6):496‐502. 25892290 10.1016/j.ctrv.2015.03.010

[11] Wang YT , Li YC , Yin LL , Pu H . Can diffusion‐weighted magnetic resonance imaging predict survival in patients with cervical cancer? A Meta‐Analysis. Eur J Radiol. 2016;85(12):2174‐2181. 27842663 10.1016/j.ejrad.2016.10.011

[12] Gillies RJ , Kinahan PE , Hricak H . Radiomics: images are more than pictures they are data. Radiology. 2016;278(2):563‐577. 26579733 10.1148/radiol.2015151169 PMC4734157

[13] Lafata KJ , Wang Y , Konkel B , Yin F‐F , Bashir MR . Radiomics: a primer on high‐throughput image phenotyping. Abdom Radiol. 2022;47(9):2986‐3002. 10.1007/s00261-021-03254-x 34435228

[14] Shur JD , Doran SJ , Kumar S , et al. Radiomics in oncology: a practical guide. Radiographics. 2021;41(6):1717‐1732. 34597235 10.1148/rg.2021210037 PMC8501897

[15] Wang T , Gao T , Guo H , et al. Preoperative prediction of parametrial invasion in early‐stage cervical cancer with MRI‐based radiomics nomogram. Eur Radiol. 2020;30(6):3585‐3593. 32065284 10.1007/s00330-019-06655-1

[16] Ren J , Li Y , Yang JJ , et al. MRI‐based radiomics analysis improves preoperative diagnostic performance for the depth of stromal invasion in patients with early stage cervical cancer. Insights Imaging. 2022;13(1):17. 35092505 10.1186/s13244-022-01156-0 PMC8800977

[17] Li Z , Li H , Wang S , et al. MR‐based radiomics nomogram of cervical cancer in prediction of the lymph‐vascular space invasion preoperatively. J Magn Reson Imaging. 2019;49(5):1420‐1426. 30362652 10.1002/jmri.26531 PMC6587470

[18] Wang S , Jiang T , Hu X , et al. Can the combination of DWI and T2WI radiomics improve the diagnostic efficiency of cervical squamous cell carcinoma? Magn Reson Imaging. 2022;92:197‐202. 35842193 10.1016/j.mri.2022.07.005

[19] Wang T , Gao T , Yang J , et al. Preoperative prediction of pelvic lymph nodes metastasis in early‐stage cervical cancer using radiomics nomogram developed based on T2‐weighted MRI and diffusion‐weighted imaging. Eur J Radiol. 2019;114:128‐135. 31005162 10.1016/j.ejrad.2019.01.003

[20] Kan Y , Dong D , Zhang Y , et al. Radiomic signature as a predictive factor for lymph node metastasis in early‐stage cervical cancer. J Magn Reson Imaging. 2019;49(1):304‐310. 30102438 10.1002/jmri.26209

[21] Hou L , Zhou W , Ren J , et al. Radiomics analysis of multiparametric MRI for the preoperative prediction of lymph node metastasis in cervical cancer. Front Oncol. 2020;10:1393. 32974143 10.3389/fonc.2020.01393 PMC7468409

[22] Zheng RR , Cai MT , Lan L , et al. An MRI‐based radiomics signature and clinical characteristics for survival prediction in early‐stage cervical cancer. Br J Radiol. 2022;95(1129):20210838. 34797703 10.1259/bjr.20210838 PMC8722251

[23] Zhou Y , Gu H‐L , Zhang X‐L , Tian Z‐F , Xu X‐Q , Tang W‐W . Multiparametric magnetic resonance imaging‐derived radiomics for the prediction of disease‐free survival in early‐stage squamous cervical cancer. Eur Radiol. 2022;32(4):2540‐2551. 34642807 10.1007/s00330-021-08326-6

[24] Fang J , Zhang B , Wang S , et al. Association of MRI‐derived radiomic biomarker with disease‐free survival in patients with early‐stage cervical cancer. Theranostics. 2020;10(5):2284‐2292. 32089742 10.7150/thno.37429 PMC7019161

[25] Zhang X , Zhao J , Zhang Q , et al. MRI‐based radiomics value for predicting the survival of patients with locally advanced cervical squamous cell cancer treated with concurrent chemoradiotherapy. Cancer Imaging. 2022;22(1):35. 35842679 10.1186/s40644-022-00474-2 PMC9287951

[26] Jiang X , Song J , Duan S , Cheng W , Chen T , Liu X . MRI radiomics combined with clinicopathologic features to predict disease‐free survival in patients with early‐stage cervical cancer. Br J Radiol. 2022;95(1136):20211229. 35604668 10.1259/bjr.20211229 PMC10162065

[27] Bhatla N , Berek JS , Cuello Fredes M , et al. Revised FIGO staging for carcinoma of the cervix uteri. Int J Gynaecol Obstet. 2019;145(1):129‐135. 30656645 10.1002/ijgo.12749

[28] Wagner‐Larsen KS , Lura N , Salvesen Ø , et al. Interobserver agreement and prognostic impact for MRI‐based 2018 FIGO staging parameters in uterine cervical cancer. Eur Radiol. 2022;32:6444‐6455. 35332408 10.1007/s00330-022-08666-x PMC9381622

[29] Yushkevich PA , Piven J , Hazlett HC , et al. User‐guided 3D active contour segmentation of anatomical structures: significantly improved efficiency and reliability. Neuroimage. 2006;31(3):1116‐1128. 16545965 10.1016/j.neuroimage.2006.01.015

[30] Cox RW , Ashburner J , Breman H , et al. A (Sort of) New Image Data Format Standard: NiFTI‐1. 2004.

[31] Andersen E . Imagedata: a python library to handle medical image data in NumPy array subclass series. J Open Source Software. 2022;7:4133.

[32] Zwanenburg A , Vallières M , Abdalah MA , et al. The image biomarker standardization initiative: standardized quantitative radiomics for high‐throughput image‐based phenotyping. Radiology. 2020;295(2):328‐338. 32154773 10.1148/radiol.2020191145 PMC7193906

[33] van Griethuysen JJM , Fedorov A , Parmar C , et al. Computational radiomics system to decode the radiographic phenotype. Cancer Res. 2017;77(21):e104‐e107. 29092951 10.1158/0008-5472.CAN-17-0339 PMC5672828

[34] Tibshirani R . The lasso method for variable selection in the Cox model. Stat Med. 1997;16(4):385‐395. 9044528 10.1002/(sici)1097-0258(19970228)16:4<385::aid-sim380>3.0.co;2-3

[35] Zou H , Hastie T . Regularization and variable selection via the elastic net. J R Stat Soc Series B Stat Methodology. 2005;67(2):301‐320.

[36] R Core Team . R: A Language and Environment for Statistical Computing. R Foundation for Statistical Computing; 2022.

[37] StataCorp . Stata Statistical Software: Release 17. StataCorp LLC; 2021.

[38] Li H , Zhu M , Jian L , et al. Radiomic score as a potential imaging biomarker for predicting survival in patients with cervical cancer. Front Oncol. 2021;11:706043. 34485139 10.3389/fonc.2021.706043 PMC8415417

[39] Cooke SL , Temple J , Macarthur S , et al. Intra‐tumour genetic heterogeneity and poor chemoradiotherapy response in cervical cancer. Br J Cancer. 2011;104(2):361‐368. 21063398 10.1038/sj.bjc.6605971 PMC3031882

[40] Fiset S , Welch ML , Weiss J , et al. Repeatability and reproducibility of MRI‐based radiomic features in cervical cancer. Radiother Oncol. 2019;135:107‐114. 31015155 10.1016/j.radonc.2019.03.001

[41] Halligan S , Menu Y , Mallett S . Why did European radiology reject my radiomic biomarker paper? How to correctly evaluate imaging biomarkers in a clinical setting. Eur Radiol. 2021;31(12):9361‐9368. 34003349 10.1007/s00330-021-07971-1 PMC8589811

