# Supplementary material for: MRI‐based radiomic signatures for pretreatment prognostication in cervical cancer
Source: Cancer Med. 2023 Oct 16;12(20):20251–65. doi: 10.1002/cam4.6526 (PMC10652318; doi:10.1002/cam4.6526)
Supplement: Supplementary file 1 — Data S1. [file CAM4-12-20251-s001.zip › cam46526-sup-0002-Tables.docx]

| **Supplementary Table 1** Clinical- and pathological characteristics for the cervical cancer patients included in this study (*n*_1_ = 133) and the entire MRI cervical cancer patient cohort (*n*_2_ = 339) diagnosed during the same period (2009–2017) at our hospital. | | | |
| --- | --- | --- | --- |
|  | Radiomics cohort  (*n*_1_ = 133) | Entire MRI cervical cancer cohort  (*n*_2_ = 339) | *p* |
| Age, median (IQR) (*n*_1_ = 133 / *n*_2_ = 339) | 48 (37–60) | 43 (36–55) | **0.02** |
| BMI, kg/m^2^, median (IQR) (*n*_1_ = 133 / *n*_2_ = 338) | 26 (22–28) | 25 (22–28) | 0.31 |
|  | *n*_1_ (%) | *n*_2_ (%) |  |
| Menopausal status (*n*_1_ = 130/*n*_2_ = 332) |  |  | **0.03** |
| Pre- /perimenopausal | 74 (57) | 226 (68) |  |
| Postmenopausal | 56 (43) | 106 (32) |  |
| MRI-derived maximum tumor size (*n*_1_ = 133/*n*_2_ = 339) |  |  | **<0.001** |
| ≤ 2 cm | 19 (14) | 155 (46) |  |
| > 2 and ≤ 4 cm | 38 (29) | 66 (20) |  |
| > 4 cm | 76 (57) | 118 (35) |  |
| 2018 FIGO stage (*n*_1_ = 133/*n*_2_ = 339) |  |  | **<0.001** |
| I | 41 (31) | 198 (58) |  |
| II | 30 (23) | 47 (14) |  |
| III | 47 (35) | 73 (20) |  |
| IV | 15 (11) | 21 (6) |  |
| Histologic type (*n*_1_ = 133/*n*_2_ = 339) |  |  | 0.58 |
| Squamous cell carcinoma | 104 (78) | 250 (74) |  |
| Adenocarcinoma | 21 (16) | 68 (20) |  |
| Other ^a^ | 8 (6) | 21 (6) |  |
| Histologic grade, (*n*_1_ = 124/*n*_2_ = 289) |  |  | 0.78 |
| 1&2 | 102 (82) | 241 (83) |  |
| 3 | 22 (18) | 48 (17) |  |
| Primary treatment (*n*_1_ = 133/*n*_2_ = 339) |  |  | **<0.001** |
| Surgery only ^b^ | 34 (26) | 173 (51) |  |
| Surgery and adjuvant treatment ^c^ | 12 (9) | 29 (9) |  |
| Radiotherapy −/+ chemotherapy | 79 (59) | 127 (38) |  |
| Other ^d^ | 8 (6) | 10 (3) |  |
| Dead from cervical cancer (*n*_1_ = 133/*n*_2_ = 339) |  |  | 0.12 |
| Yes | 33 (25) | 60 (18) |  |
| No | 100 (75) | 279 (82) |  |
| *p* values refer to Wilcoxon rank-sum test for continuous variables and Fisher’s exact test for categorical variables. Significant *p* values are given in **bold**.  BMI; body mass index; FIGO, International Federation of Gynecology and Obstetrics; IQR, interquartile range.  ^a^ Adenosquamous, neuroendocrine, and undifferentiated carcinomas.  ^b^ Conization, trachelectomy, or hysterectomy −/+ bilateral salpingectomy/salpingo-oophorectomy.  ^c^ Chemoradiation combined, chemotherapy only, or radiotherapy only.  ^d^ Neoadjuvant chemotherapy followed by surgery or palliative treatment. | | | |

| **Supplementary Table 2** Acquisition parameters for the 133 pelvic MRI examinations included in this study. Values are given as median (range) | | | | | | |
| --- | --- | --- | --- | --- | --- | --- |
|  |  | Siemens Healthineers 1.5T | GE Healthcare 1.5T | Philips Healthcare 1.5T | Siemens Healthineers 3.0T | Philips Healthcare 3.0T |
|  |  | *n* = 55 | *n* = 10 | *n* = 31 | *n* = 28 | *n* = 9 |
| T2WI TSE |  |  |  |  |  |  |
| Ax/AxObl |  |  |  |  |  |  |
|  | TR, ms | 4790 (3000–7075) | 3031 (1846–3352) | 5362 (2482–5362) | 4610 (4000–5780) | 4075 (3955–4427) |
|  | TE, ms | 100 (83–114) | 84 (79–96) | 100 (80–130) | 94 (89–97) | 110 (110–110) |
|  | FA, degree | 150 (150–180) | 160 (160–160) | 90 (90–90) | 148 (120–160) | 90 (90–90) |
|  | FOV (x,y), mm^2^ | 180x180 (160x160–200x200) | 180x180 (180x180–200x200) | 205x205 (180x180–215x215) | 200x200 (200x200–300x300) | 180x180 (180x180–180x180) |
|  | Matrix shape (x,y) | 512x512 (256x256–512x512) | 512x512 (512x512–512x512) | 512x512 (256x256–528x528) | 384x384 (320x320–384x384) | 512x512 (512x512–512x512) |
|  | Slice thickness, mm | 3.0 (3.0–5.0) | 3.0 (3.0–3.0) | 3.0 (2.5–5.0) | 3.0 (3.0–5.0) | 2.5 (2.5–2.5) |
|  | Interslice gap, mm | 0.5 (0–0.8) | 0 (0–0) | 0.3 (0–1.0) | 0.3 (0–2.0) | 0.3 (0.3–0.3) |
|  | Number of slices | 25 (15–37) | 28 (17–32) | 26 (20–40) | 24 (24–35) | 35 (30–35) |
|  | Pixel size, mm^2^ | 0.4x0.4 (0.3x0.3–0.8x0.8) | 0.4x0x4 (0.4x0.4–0.4x0.4) | 0.4x0.4 (0.3x0x3–0.8x0.8) | 0.5x0.5 (0.5x0.5–0.8x0.8) | 0.4x0x4 (0.4x0.4–0.4x0.4) |
|  | NEX | 2 (1–3) | 2 (2–2) | 6 (2–8) | 2 (2–3) | 2 (2–2) |
| DWI |  |  |  |  |  |  |
| Ax/AxObl |  |  |  |  |  |  |
|  | TR, ms | 3200 (2600–5400) | 4000 (3000–6707) | 1716 (1300–5422) | 5640 (4000–7600) | 3280 (3256–3280) |
|  | TE, ms | 82 (60–85) | 52 (51–74) | 69 (64–84) | 63 (54–78) | 85 (84–85) |
|  | FA, degree | 90 (90–90) | 90 (90–90) | 90 (90–90) | 180 (90–180) | 90 (90–90) |
|  | FOV (x,y), mm^2^ | 250x250(250x225–350x350) | 350x350 (300x300–400x400) | 375x375 (160x160–410x410) | 200x200 (200x200–360x360) | 280x280 (280x280–280x280) |
|  | Matrix shape (x,y) | 144x144(128x128–256x256) | 256x256 (256x256–256x256) | 256x256 (128x128–288x288) | 144x144 (100x100–160x160) | 352x352 (352x352–352x352) |
|  | Slice thickness, mm | 4.0 (3.0–6.0) | 5.0 (4.0–8.0) | 5.0 (4.0–7.0) | 3.0 (3.0–5.0) | 4.0 (4.0–4.0) |
|  | Interslice gap, mm | 0.6 (0.4–1.5) | 0.5 (0.4–2.0) | 1.0 (0.3–1.0) | 0.4 (0.3–3.0) | 0.4 (0.4–0.4) |
|  | Number of slices | 22 (20–30) | 24 (17–40) | 30 (14–35) | 25 (24–30) | 33 (33–33) |
|  | Pixel size, mm^2^ | 1.6x1.6 (1.0x1.0–2.7x2.7) | 1.4x1.4 (1.2x1.2–1.6x1.6) | 1.5x1.5 (1.3x1.3–2.0x2.0) | 1.4x1.4 (1.3x1.3–2.5x2.5) | 0.8x0.8 (0.8x0.8–0.8x0.8) |
|  | NEX | 10 (4–12) | 2 (1–2) | 3 (3–6) | 2 (1–12) | 2 (2–2) |
|  | b-values low, s/mm^2^ | 50 (0–50) | 0 (0–50) ^a^ | 0 (0–0) | 0 (0–50) | 0 (0–50) ^a^ |
|  | b-values high, s/mm^2^ | 800 (800–1000) | 1000 (1000–1000) ^a^ | 1000 (1000–1000) | 1000 (800–1000) | 1000 (1000–1000) ^a^ |
| Ax, axial; AxObl, axial oblique; DWI, diffusion-weighted imaging; FA, flip angle; FOV, field of view; TE, time to echo; TR, repetition time; TSE, turbo spin echo; T2WI; T2-weighted imaging; NEX, number of excitations.  ^a^ Available b-values are reported; however, some b-values were missing after the export of the image data from the scanners. | | | | | | |

| **Supplementary Table 3** List of extracted radiomic features from T2WI, high b-value DWI, and ADC maps. The reproducibility of the radiomic features extracted from the segmentations performed by both radiologists in 27 randomly chosen patients was assessed by intraclass correlation coefficients (ICCs). Only radiomic features with ICC > 0.75 were retained for further analyses (*n* = 206) (given in **bold**). | | |
| --- | --- | --- |
| Radiomic features (total *n* = 292/*n* = 206 with ICC > 0.75): | ICC | (95% CI) |
|  |  |  |
| Features from T2WI (total *n* = 106/*n* = 60 with ICC > 0.75 |  |  |
| First-order 10th Percentile T2WI | 0.61 | (0.30–0.80) |
| **First-order 90th Percentile T2WI** | **0.86** | **(0.72–0.94)** |
| **First-order Energy T2WI** | **0.98** | **(0.96–0.99)** |
| First-order Entropy T2WI | 0.75 | (0.52–0.87) |
| First-order Interquartile Range T2WI | 0.63 | (0.34–0.82) |
| First-order Kurtosis T2WI | 0.67 | (0.41–0.84) |
| **First-order Maximum T2WI** | **0.86** | **(0.72–0.93)** |
| **First-order Mean T2WI** | **0.76** | **(0.55–0.88)** |
| **First-order Mean Absolute Deviation T2WI** | **0.76** | **(0.53–0.88)** |
| **First-order Median T2WI** | **0.75** | **(0.53–0.88)** |
| First-order Minimum T2WI | 0.44 | (0.09–0.70) |
| **First-order Range T2WI** | **0.83** | **(0.66–0.92)** |
| First-order Robust Mean Absolute Deviation T2WI | 0.74 | (0.50–0.87) |
| **First-order Root Mean Squared T2WI** | **0.78** | **(0.58–0.89)** |
| **First-order Skewness T2WI** | **0.66** | **(0.38–0.83)** |
| **First-order Total Energy T2WI** | **0.99** | **(0.97–0.99)** |
| First-order Uniformity T2WI | 0.71 | (0.46–0.85) |
| First-order Variance T2WI | 0.74 | (0.48–0.87) |
| **GLCM Autocorrelation T2WI** | **0.90** | **(0.79–0.95)** |
| GLCM Cluster Prominence T2WI | 0.54 | (0.20–0.76) |
| GLCM Cluster Shade T2WI | 0.44 | (0.10–0.70) |
| GLCM Cluster Tendency T2WI | 0.76 | (0.50–0.89) |
| GLCM Contrast T2WI | 0.71 | (0.47–0.86) |
| GLCM Correlation T2WI | 0.43 | (0.08–0.69) |
| GLCM Difference Average T2WI | 0.71 | (0.45–0.86) |
| GLCM Difference Entropy T2WI | 0.69 | (0.43–0.85) |
| GLCM Difference Variance T2WI | 0.69 | (0.42–0.84) |
| GLCM Id T2WI | 0.73 | (0.48–0.87) |
| GLCM Idm T2WI | 0.72 | (0.48–0.86) |
| GLCM Idmn T2WI | 0.51 | (0.16–0.74) |
| GLCM Idn T2WI | 0.65 | (0.36–0.82) |
| GLCM Imc1 T2WI | 0.75 | (0.49–0.88) |
| GLCM Imc2 T2WI | 0.65 | (0.35–0.83) |
| GLCM Inverse Variance T2WI | 0.67 | (0.39–0.83) |
| **GLCM Joint Average T2WI** | **0.88** | **(0.76–0.94)** |
| GLCM Joint Energy T2WI | 0.75 | (0.52–0.87) |
| **GLCM Joint Entropy T2WI** | **0.76** | **(0.54–0.88)** |
| GLCM MCC T2WI | 0.52 | (0.19–0.74) |
| **GLCM Maximum Probability T2WI** | **0.75** | **(0.52–0.88)** |
| **GLCM Sum Average T2WI** | **0.88** | **(0.76–0.94)** |
| **GLCM Sum Entropy T2WI** | **0.76** | **(0.53–0.88)** |
| **GLCM Sum Squares T2WI** | **0.76** | **(0.52–0.89)** |
| GLDM Dependence Entropy T2WI | 0.74 | (0.51–0.87) |
| **GLDM Dependence Non-Uniformity T2WI** | **0.98** | **(0.96–0.99)** |
| **GLDM Dependence Non-Uniformity Normalized T2WI** | **0.91** | **(0.81–0.96)** |
| **GLDM Dependence Variance T2WI** | **0.95** | **(0.90–0.98)** |
| **GLDM Gray Level Non-Uniformity T2WI** | **0.99** | **(0.98–1.00)** |
| GLDM Gray Level Variance T2WI | 0.74 | (0.49–0.88) |
| **GLDM High Gray Level Emphasis T2WI** | **0.90** | **(0.79–0.95)** |
| **GLDM Large Dependence Emphasis T2WI** | **0.95** | **(0.89–0.98)** |
| **GLDM Large Dependence High Gray Level Emphasis T2WI** | **0.95** | **(0.90–0.98)** |
| GLDM Large Dependence Low Gray Level Emphasis T2WI | 0.62 | (0.32–0.80) |
| GLDM Low Gray Level Emphasis T2WI | 0.57 | (0.26–0.78) |
| **GLDM Small Dependence Emphasis T2WI** | **0.95** | **(0.90–0.98)** |
| **GLDM Small Dependence High Gray Level Emphasis T2WI** | **0.90** | **(0.79–0.95)** |
| GLDM Small Dependence Low Gray Level Emphasis T2WI | 0.67 | (0.40–0.83) |
| **GLRLM Gray Level Non-Uniformity T2WI** | **0.99** | **(0.98–1.00)** |
| GLRLM Gray Level Non-Uniformity Normalized T2WI | 0.71 | (0.46–0.86) |
| GLRLM Gray Level Variance T2WI | 0.74 | (0.46–0.88) |
| **GLRLM High Gray Level Run Emphasis T2WI** | **0.90** | **(0.80–0.95)** |
| **GLRLM Long Run Emphasis T2WI** | **0.91** | **(0.82–0.96)** |
| **GLRLM Long Run High Gray Level Emphasis T2WI** | **0.93** | **(0.85–0.97)** |
| GLRLM Long Run Low Gray Level Emphasis T2WI | 0.55 | (0.23–0.77) |
| GLRLM Low Gray Level Run Emphasis T2WI | 0.58 | (0.27–0.78) |
| **GLRLM Run Entropy T2WI** | **0.76** | **(0.43–0.90)** |
| **GLRLM Run Length Non-Uniformity T2WI** | **0.98** | **(0.95–0.99)** |
| GLRLM Run Length Non-Uniformity Normalized T2WI | 0.65 | (0.38–0.83) |
| GLRLM Run Percentage T2WI | 0.70 | (0.45–0.85) |
| **GLRLM Run Variance T2WI** | **0.93** | **(0.85–0.97)** |
| GLRLM Short Run Emphasis T2WI | 0.72 | (0.49–0.86) |
| **GLRLM Short Run High Gray Level Emphasis T2WI** | **0.89** | **(0.78–0.95)** |
| GLRLM Short Run Low Gray Level Emphasis T2WI | 0.59 | (0.28–0.79) |
| **GLSZM Gray Level Non-Uniformity T2WI** | **0.98** | **(0.95–0.99)** |
| GLSZM Gray Level Non-Uniformity Normalized T2WI | 0.63 | (0.34–0.81) |
| GLSZM Gray Level Variance T2WI | 0.73 | (0.46–0.87) |
| **GLSZM High Gray Level Zone Emphasis T2WI** | **0.87** | **(0.73–0.94)** |
| **GLSZM Large Area Emphasis T2WI** | **0.99** | **(0.98–1.00)** |
| **GLSZM Large Area High Gray Level Emphasis T2WI** | **0.99** | **(0.98–1.00)** |
| **GLSZM Large Area Low Gray Level Emphasis T2WI** | **0.97** | **(0.93–0.99)** |
| GLSZM Low Gray Level Zone Emphasis T2WI | 0.49 | (0.14–0.73) |
| **GLSZM Size Zone Non-Uniformity T2WI** | **0.92** | **(0.83–0.96)** |
| **GLSZM Size Zone Non-Uniformity Normalized T2WI** | **0.75** | **(0.52–0.88)** |
| GLSZM Small Area Emphasis T2WI | 0.68 | (0.42–0.84) |
| **GLSZM Small Area High Gray Level Emphasis T2WI** | **0.85** | **(0.69–0.93)** |
| GLSZM Small Area Low Gray Level Emphasis T2WI | 0.55 | (0.22–0.77) |
| **GLSZM Zone Entropy T2WI** | **0.87** | **(0.73–0.94)** |
| **GLSZM Zone Percentage T2WI** | **0.95** | **(0.89–0.98)** |
| **GLSZM Zone Variance T2WI** | **0.99** | **(0.98–1.00)** |
| **NGTDM Busyness T2WI** | **0.97** | **(0.94–0.99)** |
| NGTDM Coarseness T2WI | 0.62 | (0.32–0.80) |
| **NGTDM Complexity T2WI** | **0.86** | **(0.73–0.94)** |
| NGTDM Contrast T2WI | 0.51 | (0.17–0.74) |
| NGTDM Strength T2WI | 0.72 | (0.46–0.86) |
| **Shape Elongation T2WI** | **0.83** | **(0.67–0.92)** |
| **Shape Flatness T2WI** | **0.89** | **(0.78–0.95)** |
| **Shape Least Axis Length T2WI** | **0.96** | **(0.92–0.98)** |
| **Shape Major Axis Length T2WI** | **0.98** | **(0.97–0.99)** |
| **Shape Maximum 2D Diameter Column T2WI** | **0.98** | **(0.96–0.99)** |
| **Shape Maximum 2D Diameter Row T2WI** | **0.98** | **(0.96–0.99)** |
| **Shape Maximum 2D Diameter Slice T2WI** | **0.98** | **(0.95–0.99)** |
| **Shape Maximum 3D Diameter T2WI** | **0.98** | **(0.96–0.99)** |
| **Shape Mesh Volume T2WI** | **1.00** | **(0.99–1.00)** |
| **Shape Minor Axis Length T2WI** | **0.98** | **(0.95–0.99)** |
| **Shape Sphericity T2WI** | **0.81** | **(0.34–0.93)** |
| **Shape Surface Area T2WI** | **0.94** | **(0.88–0.97)** |
| **Shape Surface Volume Ratio T2WI** | **0.89** | **(0.78–0.95)** |
|  |  |  |
| Features from high b-value DWI (total *n* = 93/*n* = 77 with ICC > 0.75) |  |  |
| **First-order 10th Percentile DWI** | **0.84** | **(0.67–0.92)** |
| **First-order 90th Percentile DWI** | **0.98** | **(0.95–0.99)** |
| **First-order Energy DWI** | **0.99** | **(0.99–1.00)** |
| **First-order Entropy DWI** | **0.87** | **(0.70–0.94)** |
| **First-order Interquartile Range DWI** | **0.87** | **(0.74–0.94)** |
| First-order Kurtosis DWI | 0.53 | (0.19–0.76) |
| **First-order Maximum DWI** | **0.95** | **(0.88–0.97)** |
| **First-order Mean DWI** | **0.94** | **(0.87–0.97)** |
| **First-order Mean Absolute Deviation DWI** | **0.93** | **(0.85–0.97)** |
| **First-order Median DWI** | **0.92** | **(0.84–0.96)** |
| First-order Minimum DWI | 0.71 | (0.45–0.85) |
| **First-order Range DWI** | **0.95** | **(0.88–0.97)** |
| **First-order Robust Mean Absolute Deviation DWI** | **0.89** | **(0.78–0.95)** |
| **First-order Root Mean Squared DWI** | **0.95** | **(0.90–0.98)** |
| First-order Skewness DWI | 0.67 | (0.41–0.84) |
| **First-order Total Energy DWI** | **1.00** | **(0.99–1.00)** |
| First-order Uniformity DWI | 0.73 | (0.46–0.87) |
| **First-order Variance DWI** | **0.95** | **(0.89–0.98)** |
| **GLCM Autocorrelation DWI** | **0.95** | **(0.89–0.98)** |
| **GLCM Cluster Prominence DWI** | **0.92** | **(0.83–0.96)** |
| **GLCM Cluster Shade DWI** | **0.85** | **(0.70–0.93)** |
| **GLCM Cluster Tendency DWI** | **0.92** | **(0.82–0.96)** |
| **GLCM Contrast DWI** | **0.95** | **(0.89–0.98)** |
| GLCM Correlation DWI | 0.53 | (0.19–0.75) |
| **GLCM Difference Average DWI** | **0.93** | **(0.86–0.97)** |
| **GLCM Difference Entropy DWI** | **0.93** | **(0.85–0.97)** |
| **GLCM Difference Variance DWI** | **0.98** | **(0.96–0.99)** |
| **GLCM Id DWI** | **0.87** | **(0.73–0.94)** |
| **GLCM Idm DWI** | **0.87** | **(0.73–0.94)** |
| **GLCM Idmn DWI** | **0.89** | **(0.78–0.95)** |
| **GLCM Idn DWI** | **0.84** | **(0.69–0.93)** |
| GLCM Imc1 DWI | 0.47 | (0.14–0.72) |
| GLCM Imc2 DWI | 0.49 | (0.16–0.73) |
| GLCM Inverse Variance DWI | 0.57 | (0.26–0.78) |
| **GLCM Joint Average DWI** | **0.93** | **(0.86–0.97)** |
| **GLCM Joint Energy DWI** | **0.89** | **(0.75–0.95)** |
| **GLCM Joint Entropy DWI** | **0.92** | **(0.82–0.97)** |
| GLCM MCC DWI | 0.51 | (0.17–0.74) |
| **GLCM Maximum Probability DWI** | **0.95** | **(0.87–0.98)** |
| **GLCM Sum Average DWI** | **0.93** | **(0.86–0.97)** |
| **GLCM Sum Entropy DWI** | **0.87** | **(0.70–0.94)** |
| **GLCM Sum Squares DWI** | **0.94** | **(0.87–0.97)** |
| **GLDM Dependence Entropy DWI** | **0.97** | **(0.93–0.98)** |
| **GLDM Dependence Non-Uniformity DWI** | **0.98** | **(0.95–0.99)** |
| **GLDM Dependence Non-Uniformity Normalized DWI** | **0.85** | **(0.69–0.93)** |
| **GLDM Dependence Variance DWI** | **0.85** | **(0.70–0.93)** |
| **GLDM Gray Level Non-Uniformity DWI** | **0.97** | **(0.94–0.99)** |
| **GLDM Gray Level Variance DWI** | **0.95** | **(0.89–0.98)** |
| **GLDM High Gray Level Emphasis DWI** | **0.95** | **(0.89–0.98)** |
| **GLDM Large Dependence Emphasis DWI** | **0.92** | **(0.82–0.96)** |
| **GLDM Large Dependence High Gray Level Emphasis DWI** | **0.96** | **(0.92–0.98)** |
| GLDM Large Dependence Low Gray Level Emphasis DWI | 0.58 | (0.26–0.78) |
| **GLDM Low Gray Level Emphasis DWI** | **0.78** | **(0.58–0.90)** |
| **GLDM Small Dependence Emphasis DWI** | **0.94** | **(0.87–0.97)** |
| **GLDM Small Dependence High Gray Level Emphasis DWI** | **0.95** | **(0.90–0.98)** |
| **GLDM Small Dependence Low Gray Level Emphasis DWI** | **0.77** | **(0.57–0.89)** |
| **GLRLM Gray Level Non-Uniformity DWI** | **0.97** | **(0.94–0.99)** |
| GLRLM Gray Level Non-Uniformity Normalized DWI | 0.70 | (0.41–0.85) |
| **GLRLM Gray Level Variance DWI** | **0.95** | **(0.90–0.98)** |
| **GLRLM High Gray Level Run Emphasis DWI** | **0.95** | **(0.89–0.98)** |
| **GLRLM Long Run Emphasis DWI** | **0.88** | **(0.75–0.94)** |
| **GLRLM Long Run High Gray Level Emphasis DWI** | **0.89** | **(0.78–0.95)** |
| **GLRLM Long Run Low Gray Level Emphasis DWI** | **0.93** | **(0.86–0.97)** |
| **GLRLM Low Gray Level Run Emphasis DWI** | **0.83** | **(0.66–0.92)** |
| GLRLM Run Entropy DWI | 0.67 | (0.40–0.84) |
| **GLRLM Run Length Non-Uniformity DWI** | **0.98** | **(0.96–0.99)** |
| **GLRLM Run Length Non-Uniformity Normalized DWI** | **0.76** | **(0.53–0.88)** |
| GLRLM Run Percentage DWI | 0.73 | (0.48–0.87) |
| **GLRLM Run Variance DWI** | **0.90** | **(0.79–0.95)** |
| GLRLM Short Run Emphasis DWI | 0.71 | (0.45–0.86) |
| **GLRLM Short Run High Gray Level Emphasis DWI** | **0.95** | **(0.89–0.98)** |
| GLRLM Short Run Low Gray Level Emphasis DWI | 0.58 | (0.26–0.79) |
| **GLSZM Gray Level Non-Uniformity DWI** | **0.97** | **(0.93–0.99)** |
| **GLSZM Gray Level Non-Uniformity Normalized DWI** | **0.91** | **(0.81–0.96)** |
| **GLSZM Gray Level Variance DWI** | **0.98** | **(0.95–0.99)** |
| **GLSZM High Gray Level Zone Emphasis DWI** | **0.94** | **(0.87–0.97)** |
| **GLSZM Large Area Emphasis DWI** | **0.98** | **(0.95–0.99)** |
| **GLSZM Large Area High Gray Level Emphasis DWI** | **0.97** | **(0.93–0.98)** |
| **GLSZM Large Area Low Gray Level Emphasis DWI** | **0.93** | **(0.85–0.97)** |
| **GLSZM Low Gray Level Zone Emphasis DWI** | **0.86** | **(0.72–0.93)** |
| **GLSZM Size Zone Non-Uniformity DWI** | **0.95** | **(0.90–0.98)** |
| **GLSZM Size Zone Non-Uniformity Normalized DWI** | **0.95** | **(0.90–0.98)** |
| **GLSZM Small Area Emphasis DWI** | **0.94** | **(0.87–0.97)** |
| **GLSZM Small Area High Gray Level Emphasis DWI** | **0.95** | **(0.89–0.98)** |
| **GLSZM Small Area Low Gray Level Emphasis DWI** | **0.77** | **(0.56–0.89)** |
| **GLSZM Zone Entropy DWI** | **0.93** | **(0.86–0.97)** |
| **GLSZM Zone Percentage DWI** | **0.93** | **(0.85–0.97)** |
| **GLSZM Zone Variance DWI** | **0.98** | **(0.95–0.99)** |
| **NGTDM Busyness DWI** | **0.98** | **(0.95–0.99)** |
| NGTDM Coarseness DWI | 0.65 | (0.37–0.82) |
| **NGTDM Complexity DWI** | **0.98** | **(0.96–0.99)** |
| **NGTDM Contrast DWI** | **0.91** | **(0.79–0.96)** |
| **NGTDM Strength DWI** | **0.90** | **(0.80–0.95)** |
|  |  |  |
| Features from ADC (total *n* = 93/*n* = 69 with ICC > 0.75) |  |  |
| **First-order 10th Percentile ADC** | **0.89** | **(0.77–0.95)** |
| **First-order 90th Percentile ADC** | **0.84** | **(0.68–0.93)** |
| **First-order Energy ADC** | **0.92** | **(0.84–0.97)** |
| **First-order Entropy ADC** | **0.86** | **(0.72–0.93)** |
| First-order Interquartile Range ADC | 0.72 | (0.48–0.86) |
| First-order Kurtosis ADC | 0.70 | (0.43–0.85) |
| **First-order Maximum ADC** | **0.81** | **(0.62–0.91)** |
| **First-order Mean ADC** | **0.89** | **(0.77–0.95)** |
| **First-order Mean Absolute Deviation ADC** | **0.76** | **(0.53–0.88)** |
| **First-order Median ADC** | **0.88** | **(0.76–0.94)** |
| First-order Minimum ADC | 0.73 | (0.50–0.87) |
| **First-order Range ADC** | **0.87** | **(0.74–0.94)** |
| **First-order Robust Mean Absolute Deviation ADC** | **0.78** | **(0.57–0.89)** |
| **First-order Root Mean Squared ADC** | **0.87** | **(0.74–0.94)** |
| **First-order Skewness ADC** | **0.81** | **(0.63–0.91)** |
| **First-order Total Energy ADC** | **0.89** | **(0.77–0.95)** |
| **First-order Uniformity ADC** | **0.91** | **(0.81–0.96)** |
| First-order Variance ADC | 0.62 | (0.32–0.81) |
| **GLCM Autocorrelation ADC** | **0.81** | **(0.62–0.91)** |
| GLCM Cluster Prominence ADC | 0.41 | (0.04–0.68) |
| **GLCM Cluster Shade ADC** | **0.87** | **(0.75–0.94)** |
| GLCM Cluster Tendency ADC | 0.53 | (0.19–0.76) |
| **GLCM Contrast ADC** | **0.88** | **(0.75–0.94)** |
| GLCM Correlation ADC | 0.54 | (0.20–0.76) |
| **GLCM Difference Average ADC** | **0.77** | **(0.56–0.89)** |
| **GLCM Difference Entropy ADC** | **0.81** | **(0.62–0.91)** |
| **GLCM Difference Variance ADC** | **0.93** | **(0.84–0.97)** |
| **GLCM Id ADC** | **0.76** | **(0.54–0.88)** |
| **GLCM Idm ADC** | **0.76** | **(0.54–0.88)** |
| GLCM Idmn ADC | 0.72 | (0.48–0.86) |
| GLCM Idn ADC | 0.67 | (0.39–0.83) |
| GLCM Imc1 ADC | 0.64 | (0.35–0.82) |
| GLCM Imc2 ADC | 0.69 | (0.42–0.85) |
| GLCM Inverse Variance ADC | 0.62 | (0.34–0.81) |
| **GLCM Joint Average ADC** | **0.87** | **(0.72–0.94)** |
| **GLCM Joint Energy ADC** | **0.87** | **(0.74–0.94)** |
| **GLCM Joint Entropy ADC** | **0.78** | **(0.57–0.89)** |
| GLCM MCC ADC | 0.68 | (0.41–0.84) |
| **GLCM Maximum Probability ADC** | **0.82** | **(0.65–0.92)** |
| **GLCM Sum Average ADC** | **0.87** | **(0.72–0.94)** |
| **GLCM Sum Entropy ADC** | **0.87** | **(0.74–0.94)** |
| GLCM Sum Squares ADC | 0.56 | (0.23–0.77) |
| **GLDM Dependence Entropy ADC** | **0.83** | **(0.66–0.92)** |
| **GLDM Dependence Non-Uniformity ADC** | **0.98** | **(0.95–0.99)** |
| **GLDM Dependence Non-Uniformity Normalized ADC** | **0.86** | **(0.72–0.93)** |
| **GLDM Dependence Variance ADC** | **0.95** | **(0.89–0.98)** |
| **GLDM Gray Level Non-Uniformity ADC** | **0.99** | **(0.98–1.00)** |
| GLDM Gray Level Variance ADC | 0.62 | (0.31–0.81) |
| **GLDM High Gray Level Emphasis ADC** | **0.83** | **(0.66–0.92)** |
| **GLDM Large Dependence Emphasis ADC** | **0.97** | **(0.94–0.99)** |
| **GLDM Large Dependence High Gray Level Emphasis ADC** | **0.92** | **(0.83–0.96)** |
| **GLDM Large Dependence Low Gray Level Emphasis ADC** | **0.95** | **(0.90–0.98)** |
| **GLDM Low Gray Level Emphasis ADC** | **0.88** | **(0.75–0.94)** |
| **GLDM Small Dependence Emphasis ADC** | **0.89** | **(0.77–0.95)** |
| GLDM Small Dependence High Gray Level Emphasis ADC | 0.62 | (0.33–0.81) |
| **GLDM Small Dependence Low Gray Level Emphasis ADC** | **0.76** | **(0.54–0.88)** |
| **GLRLM Gray Level Non-Uniformity ADC** | **0.98** | **(0.96–0.99)** |
| **GLRLM Gray Level Non-Uniformity Normalized ADC** | **0.92** | **(0.82–0.96)** |
| GLRLM Gray Level Variance ADC | 0.60 | (0.29–0.80) |
| **GLRLM High Gray Level Run Emphasis ADC** | **0.83** | **(0.66–0.92)** |
| **GLRLM Long Run Emphasis ADC** | **0.86** | **(0.73–0.94)** |
| **GLRLM Long Run High Gray Level Emphasis ADC** | **0.89** | **(0.76–0.95)** |
| **GLRLM Long Run Low Gray Level Emphasis ADC** | **0.81** | **(0.64–0.91)** |
| **GLRLM Low Gray Level Run Emphasis ADC** | **0.88** | **(0.76–0.95)** |
| **GLRLM Run Entropy ADC** | **0.91** | **(0.81–0.96)** |
| **GLRLM Run Length Non-Uniformity ADC** | **0.97** | **(0.93–0.99)** |
| GLRLM Run Length Non-Uniformity Normalized ADC | 0.71 | (0.46–0.85) |
| GLRLM Run Percentage ADC | 0.72 | (0.47–0.86) |
| **GLRLM Run Variance ADC** | **0.87** | **(0.73–0.94)** |
| GLRLM Short Run Emphasis ADC | 0.73 | (0.49–0.87) |
| **GLRLM Short Run High Gray Level Emphasis ADC** | **0.82** | **(0.63–0.91)** |
| **GLRLM Short Run Low Gray Level Emphasis ADC** | **0.90** | **(0.79–0.95)** |
| **GLSZM Gray Level Non-Uniformity ADC** | **0.77** | **(0.55–0.89)** |
| **GLSZM Gray Level Non-Uniformity Normalized ADC** | **0.93** | **(0.86–0.97)** |
| **GLSZM Gray Level Variance ADC** | **0.83** | **(0.66–0.92)** |
| **GLSZM High Gray Level Zone Emphasis ADC** | **0.85** | **(0.70–0.93)** |
| **GLSZM Large Area Emphasis ADC** | **0.92** | **(0.84–0.96)** |
| **GLSZM Large Area High Gray Level Emphasis ADC** | **0.94** | **(0.87–0.97)** |
| **GLSZM Large Area Low Gray Level Emphasis ADC** | **0.90** | **(0.80–0.96)** |
| **GLSZM Low Gray Level Zone Emphasis ADC** | **0.85** | **(0.70–0.93)** |
| GLSZM Size Zone Non-Uniformity ADC | 0.70 | (0.44–0.85) |
| **GLSZM Size Zone Non-Uniformity Normalized ADC** | **0.84** | **(0.67–0.92)** |
| **GLSZM Small Area Emphasis ADC** | **0.82** | **(0.65–0.92)** |
| **GLSZM Small Area High Gray Level Emphasis ADC** | **0.77** | **(0.55–0.89)** |
| GLSZM Small Area Low Gray Level Emphasis ADC | 0.52 | (0.18–0.75) |
| **GLSZM Zone Entropy ADC** | **0.87** | **(0.67–0.94)** |
| **GLSZM Zone Percentage ADC** | **0.84** | **(0.68–0.92)** |
| **GLSZM Zone Variance ADC** | **0.92** | **(0.84–0.96)** |
| **NGTDM Busyness ADC** | **0.84** | **(0.68–0.92)** |
| NGTDM Coarseness ADC | 0.58 | (0.28–0.78) |
| NGTDM Complexity ADC | 0.87 | (0.74–0.94) |
| NGTDM Contrast ADC | 0.88 | (0.76–0.94) |
| NGTDM Strength ADC | 0.50 | (0.15–0.74) |
| ADC, Apparent diffusion coefficient; DWI, Diffusion-weighted imaging; GLCM, Gray Level Co-occurrence Matrix; GLDM, Gray Level Dependence Matrix; GLRLM, Gray Level Run Length Matrix; GLSZM, Gray Level Size Zone Matrix; NGTDM, Neighboring Gray Tone Difference Matrix; T2WI, T2-weighted imaging. | | |

| **Supplementary Table 4** Radiomic feature coefficients selected by LASSO Cox regression and Elastic net Cox regression from T2WI alone and T2WI in combination with DWI for prediction of disease-specific survival (DSS) in cervical cancer. The regularization parameter (λ) was optimized by leave-one-out cross-validation, and the parameter α was set to 0.5. The radiomic signatures were derived from the training cohort (*n*_T_ = 89) and tested in the validation cohort (*n*_V_ = 44). The performance metrics of the radiomic signatures for predicting DSS are given as AUC (5-year DSS) and C-index (overall DSS). | | | | |
| --- | --- | --- | --- | --- |
| Radiomic features: | Radiomic signature T2_rad_ ^a^ | | Radiomic signature T2+DWI_rad_ ^b^ | |
|  | LASSO Cox | Elastic net Cox | LASSO Cox | Elastic net Cox |
| GLSZM Large Area Low Gray Level Emphasis T2WI | 0.188 | 0.153 | 0.276 | 0.176 |
| Shape Major Axis Length T2WI | 0.333 | 0.131 |  | 0.109 |
| Shape Maximum 2D Diameter Column T2WI |  | 0.053 |  | 0.032 |
| Shape Maximum 2D Diameter Row T2WI |  | 0.023 |  |  |
| Shape Maximum 2D Diameter Slice T2WI | 0.003 | 0.034 |  | 0.002 |
| Shape Maximum 3D Diameter T2WI |  | 0.048 |  | 0.028 |
| Shape Minor Axis Length T2WI |  | 0.003 |  |  |
| Shape Surface Area T2WI | 0.093 | 0.122 | 0.291 | 0.149 |
| GLCM Cluster Shade DWI (high b-value) |  |  | 0.001 |  |
| GLSZM Size Zone Non-Uniformity DWI (high b-value) |  |  | 0.173 | 0.078 |
| GLSZM Gray Level Non-Uniformity ADC |  |  | 0.276 | 0.152 |
|  |  |  |  |  |
| Regularization parameters λ / α | 0.112 | 0.223/0.5 | 0.097 | 0.214/0.5 |
| Metrics for prognostic performance | Radiomic signature T2_rad_ ^a^ | | Radiomic signature T2+DWI_rad_ ^b^ | |
| (*n*_T_ = 89/*n*_V_ = 44) | LASSO Cox | Elastic net Cox | LASSO Cox | Elastic net Cox |
| AUC_T_ | 0.80 | 0.79 | 0.81 | 0.81 |
| AUC_V_ | 0.62 | 0.62 | 0.75 | 0.70 |
| C-index_T_ | 0.73 | 0.73 | 0.76 | 0.75 |
| C-index_V_ | 0.63 | 0.64 | 0.72 | 0.69 |
| ADC, Apparent diffusion coefficient; AUC, Area under the time-dependent receiver operating characteristic (tdROC) curves; C-Index, Concordance Index; DWI, Diffusion-weighted imaging; GLCM, Gray Level Co-occurrence Matrix; GLSZM, Gray Level Size Zone Matrix; LASSO, Least absolute shrinkage and selection operator; T2WI, T2-weighted imaging.  ^a^ Radiomic features derived only from T2WI.  ^b^ Radiomic features derived from T2WI and DWI (high b-value and ADC). | | | | |

| **Supplementary Table 5** Correlation (Spearman correlation coefficients, *r*_S_) between the radiomic features in T2_rad_ | | | | |
| --- | --- | --- | --- | --- |
| GLSZM Large Area Low Gray Level Emphasis T2WI | 1.0 |  |  |  |
| Shape Major Axis Length T2WI | 0.58* | 1.0 |  |  |
| Shape Maximum 2D Diameter Slice T2WI | 0.51* | 0.76* | 1.0 |  |
| Shape Surface Area T2WI | 0.58* | 0.88* | 0.94* | 1.0 |
|  | GLSZM Large Area Low Gray Level Emphasis T2WI | Shape Major Axis Length T2WI | Shape Maximum 2D Diameter Slice T2WI | Shape Surface Area T2WI |
| *Correlation is significant at the 0.05 level.  GLSZM, Gray Level Size Zone Matrix; T2WI, T2-weighted imaging. | | | | |

| **Supplementary Table 6** Correlation (Spearman correlation coefficients, *r*_S_) between the radiomic features in T2+DWI_rad_ | | | | | |
| --- | --- | --- | --- | --- | --- |
| GLSZM Large Area Low Gray Level Emphasis T2WI | 1.0 |  |  |  |  |
| Shape Surface Area T2WI | 0.58* | 1.0 |  |  |  |
| GLCM Cluster Shade DWI | 0.03 | 0.10 | 1.0 |  |  |
| GLSZM Size Zone Non-Uniformity DWI | 0.41* | 0.50* | −0.07 | 1.0 |  |
| GLSZM Gray Level Non-Uniformity ADC | 0.55* | 0.78* | 0.09 | 0.38* | 1.0 |
|  | GLSZM Large Area Low Gray Level Emphasis T2WI | Shape Surface Area T2WI | GLCM Cluster Shade DWI | GLSZM Size Zone Non-Uniformity DWI | GLSZM Gray Level Non-Uniformity ADC |
| *Correlation is significant at the 0.05 level.  ADC, Apparent diffusion coefficient; DWI, Diffusion-weighted imaging; GLCM, Gray Level Co-occurrence Matrix; GLSZM, Gray Level Size Zone Matrix; T2WI, T2-weighted imaging. | | | | | |

| **Supplementary Table 7** The performance of models combining MRI-derived maximum tumor size ≤/> 4 cm (MAX_size_) with the radiomic signatures (MAX_size_-radiomic models) and MAX_size_ alone for predicting 5-year disease-specific survival (DSS) in cervical cancer. | | | | |
| --- | --- | --- | --- | --- |
|  | Training cohort (*n*_T_ = 89) | | Validation cohort (*n*_V_ = 44) | |
|  | AUC_T_ | AIC_T_ | AUC_V_ | AIC_V_ |
| MAX_size_ with T2_rad_ | 0.81 | 181.6 | 0.63 | 66.3 |
| MAX_size_ with T2+DWI_rad_ | 0.82 | 172.2 | 0.74 | 66.5 |
| MAX_size_ | 0.69 | 193.3 | 0.65 | 66.4 |
| AUC, Area under the time-dependent receiver operating characteristic (tdROC) curves; AIC, Akaike information criterion. | | | | |

| **Supplementary Table 8** Patient outcomes (disease progression and disease-specific death) in patients with low- and high T2_rad_ and T2+DWI_rad_ radiomic scores. | | | | | | |
| --- | --- | --- | --- | --- | --- | --- |
|  | T2_rad_  *n* (%) | |  | T2+DWI_rad_  *n* (%) | |  |
|  | Low  (*n* = 93) | High  (*n* = 40) | *p* | Low  (*n* = 101) | High  (*n* = 32) | *p* |
| Progression ^a^ |  |  | **0.003** |  |  | **<0.001** |
| No (*n* = 95) | 74 (80) | 21 (53) |  | 82 (81) | 13 (41) |  |
| Yes (*n* = 38) | 19 (20) | 19 (48) |  | 19 (19) | 19 (59) |  |
| Progression ^a^ |  |  | **0.003** |  |  | **<0.001** |
| No (*n* = 95) | 74 (80) | 21 (53) |  | 82 (81) | 13 (41) |  |
| Local ^b^ (*n* = 16) | 10 (11) | 6 (15) |  | 10 (10) | 6 (19) |  |
| Distant ^c^ (*n* = 22) | 9 (10) | 13 (33) |  | 9 (9) | 13 (41) |  |
| Disease-specific death |  |  | **<0.001** |  |  | **<0.001** |
| No (*n* = 100) | 79 (85) | 21 (53) |  | 87 (86) | 13 (41) |  |
| Yes (*n* = 33) | 14 (15) | 19 (48) |  | 14 (14) | 19 (59) |  |
| *p* values refer to Fischer’s exact test. Significant *p* values are given in **bold**.  ^a^ Verified by clinical examination with biopsies or imaging methods like computer tomography (CT), MRI, and/or ^18^F-fluorodeoxyglucose positron emission tomography with CT (FDG-PET/CT).  ^b^ Recurrence or progression within the pelvis.  ^c^ New metastases in the abdomen or at distant sites. | | | | | | |
