# Supplementary material for: MRI‐based radiomic signatures for pretreatment prognostication in cervical cancer
Source: Cancer Med. 2023 Oct 16;12(20):20251–65. doi: 10.1002/cam4.6526 (PMC10652318; doi:10.1002/cam4.6526)
Supplement: Supplementary file 1 — Data S1. [file CAM4-12-20251-s001.zip › cam46526-sup-0003-AppendixS1.docx]

**Supplementary Appendix 1**

LASSO Cox and Elastic net Cox radiomic signature calculation formulas:

**A.** LASSO Cox radiomic signature T2_rad_ =

(0.188 x GLSZM Large Area Low Gray Level Emphasis T2WI)

+ (0.333 x Shape Major Axis Length T2WI)

+ (0.003 x Shape Maximum 2D Diameter Slice T2WI)

+ (0.093 x Shape Surface Area T2WI)

**B.** LASSO Cox radiomic signature T2+DWI_rad_ =

(0.276 x GLSZM Large Area Low Gray Level Emphasis T2WI)

+ (0.291 x Shape Surface Area T2WI)

+ (0.001 x GLCM Cluster Shade DWI (high b-value))

+ (0.173 x GLSZM Size Zone Non-Uniformity DWI (high b-value))

+ (0.276 x GLSZM Gray Level Non-Uniformity ADC)

**C.** Elastic net Cox radiomic signature T2_rad_ =

(0.153 x GLSZM Large Area Low Gray Level Emphasis T2WI)

+ (0.131 x Shape Major Axis Length T2WI)

+ (0.053 x Shape Maximum 2D Diameter Column T2WI)

+ (0.023 x Shape Maximum 2D Diameter Row T2WI)

+ (0.034 x Shape Maximum 2D Diameter Slice T2WI)

+ (0.048 x Shape Maximum 3D Diameter T2WI)

+ (0.003 x Shape Minor Axis Length T2WI)

+ (0.122 x Shape Surface Area T2WI)

**D.** Elastic net Cox radiomic signature T2+DWI_rad_ =

(0.176 x GLSZM Large Area Low Gray Level Emphasis T2WI)

+ (0.109 x Shape Major Axis Length T2WI)

+ (0.032 x Shape Maximum 2D Diameter Column T2WI)

+ (0.002 x Shape Maximum 2D Diameter Slice T2WI)

+ (0.028 x Shape Maximum 3D Diameter T2WI)

+ (0.149 x Shape Surface Area T2WI)

+ (0.078 x GLSZM Size Zone Non-Uniformity DWI (high b-value))

+ (0.152 x GLSZM Gray Level Non-Uniformity ADC)
