# Supplementary material for: MRI‐based radiomic signatures for pretreatment prognostication in cervical cancer
Source: Cancer Med. 2023 Oct 16;12(20):20251–65. doi: 10.1002/cam4.6526 (PMC10652318; doi:10.1002/cam4.6526)
Supplement: Supplementary file 1 — Data S1. [file CAM4-12-20251-s001.zip › cam46526-sup-0001-FigureS1.docx]

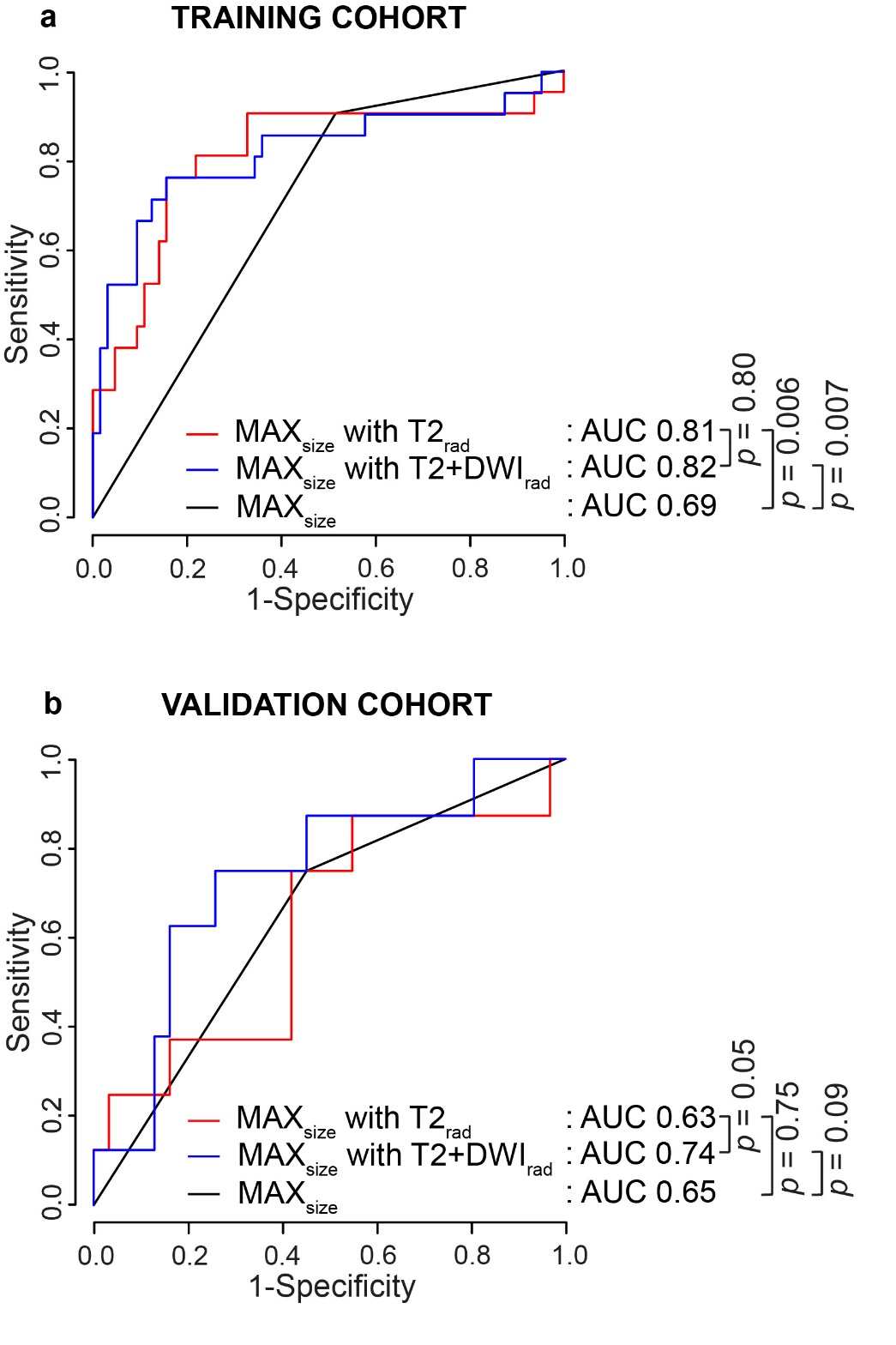


**Supplementary Figure 1** Time-dependent receiver operating characteristic (tdROC) curves for prediction of 5-year disease-specific survival (DSS) based on MRI-derived maximum tumor size ≤/> 4 cm (MAX_size_), a combined model of MAX_size_ with T2_rad_, and a combined model of MAX_size_ with T2+DWI_rad_ in the training (*n*_T_ = 89) (**a**) and the validation (*n*_V_ = 44) (**b**) cohorts. *p* values refer to the test of equal area under the tdROC curves (AUC).
