# Supplementary figures and images for: MRI‐based radiomic signatures for pretreatment prognostication in cervical cancer
Source: Cancer Med. 2023 Oct 16;12(20):20251–65. doi: 10.1002/cam4.6526 (PMC10652318; doi:10.1002/cam4.6526)

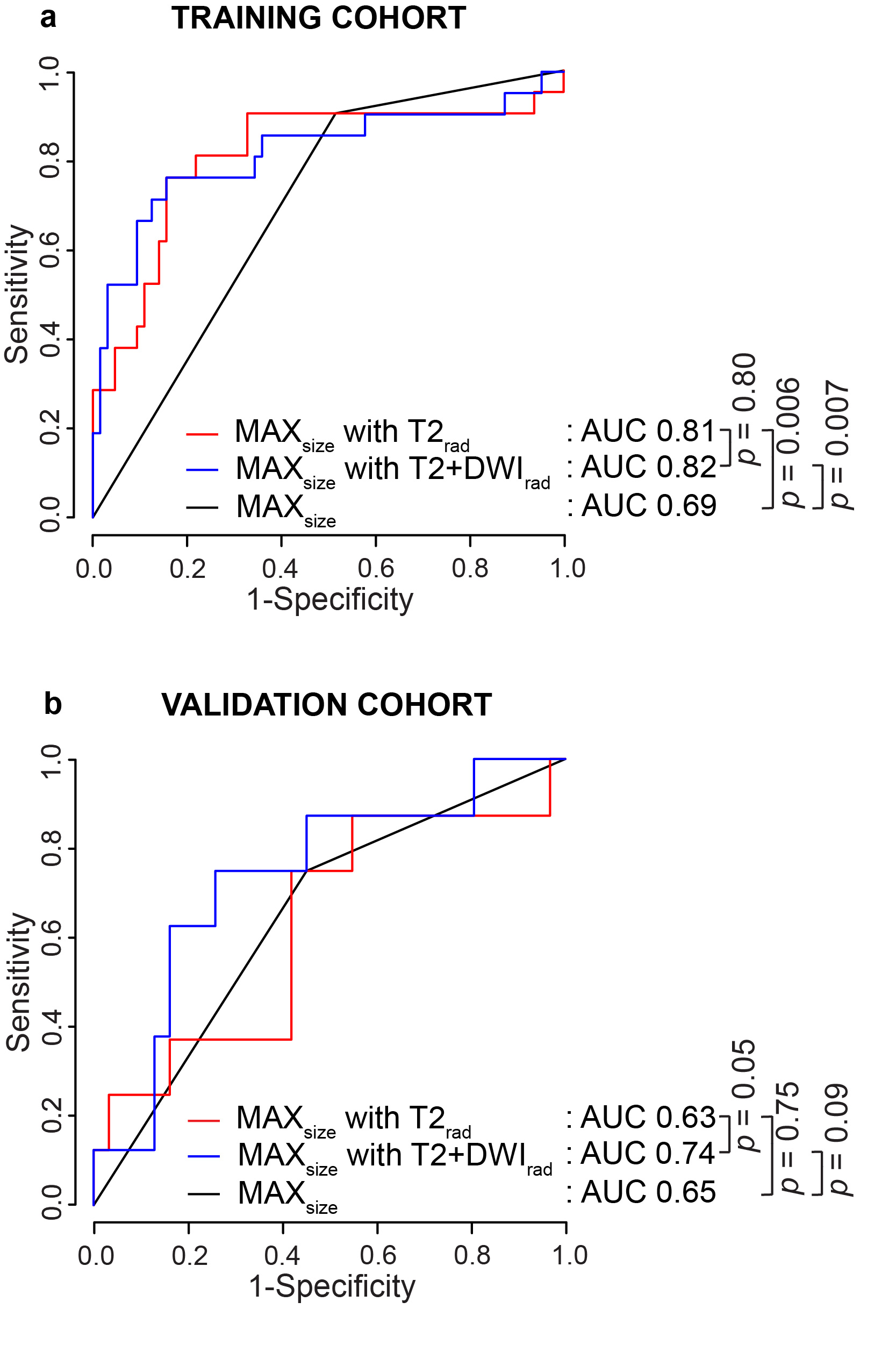

Supplement: Supplementary file 1 — Data S1. [file CAM4-12-20251-s001.zip › cam46526-sup-0001-FigureS1.jpg.jpg]
